# Association between bacterial vaginosis prevalence and maternal characteristics in pregnant women: Diagnostic comparison of BV Rapid Blue and Amsel’s criteria

**DOI:** 10.1371/journal.pone.0355948

**Published:** 2026-08-14

**Authors:** Harya Hermawan, Dhanny Primantara Johari Santoso, Annisa Dewi Nugrahani, Muhammad Alamsyah Aziz

**Affiliations:** 1 Department of Obstetrics and Gynecology, Faculty of Medicine, Universitas Padjadjaran, Java, Indonesia; 2 Doctoral Program, Faculty of Medicine, Universitas Padjadjaran, Java, Indonesia; University of Idaho, UNITED STATES OF AMERICA

## Abstract

**Background:**

Bacterial vaginosis (BV) is a common vaginal infection associated with adverse pregnancy outcomes and increased susceptibility to sexually transmitted infections. This study evaluated the diagnostic performance of the BV Rapid Blue point-of-care test compared with Amsel’s criteria and assessed maternal characteristics and pregnancy outcomes among BV-positive pregnant women at Dr. Slamet Regional Hospital, Garut.

**Method:**

A cross-sectional diagnostic accuracy study was conducted among 131 pregnant women between April and May 2025. The diagnostic performance of BV Rapid Blue was evaluated using Amsel’s criteria as the pragmatic clinical reference standard. Diagnostic accuracy parameters were calculated, and multivariate logistic regression was performed to identify factors associated with BV.

**Results:**

BV prevalence was 61.1%. Most participants were aged 25–35, multigravida, and had high school education. All participants reported vaginal discharge, while only 3.1% reported a history of STIs. Common complications included preeclampsia (46.6%) and PROM (32.1%). BV Rapid Blue showed strong agreement with Amsel’s criteria (p < 0.0001), with sensitivity 95.4%, specificity 55.7%, PPV 51.3%, and NPV 96.1%. BV recurrence (OR = 4.29, p < 0.0001) and secondary infection (OR = 2.56, p = 0.01) were significant predictors, while higher education was protective (OR = 0.52, p = 0.02).

**Conclusions:**

BV remains prevalent in pregnancy. BV Rapid Blue is a reliable, rapid diagnostic tool with high sensitivity and NPV. Early diagnosis may facilitate earlier clinical assessment and management, particularly in settings where laboratory-based diagnostics are limited.

## Introduction

Bacterial Vaginosis (BV) is a clinical condition characterized by a disruption in the normal vaginal microbiota, marked by a significant reduction in *Lactobacillus* species and an overgrowth of anaerobic bacteria [1]. Previously referred to as *Gardnerella* vaginitis—implying a singular pathogenic role of *Gardnerella vaginalis*—the term “Bacterial Vaginosis” more accurately reflects the polymicrobial etiology of this condition, involving a complex array of anaerobes such as *Atopobium vaginae*, *Mobiluncus spp.*, and *Prevotella spp.* [1–5]. This redefinition underscores the evolving understanding of BV as a dysbiosis-related syndrome rather than a classic infection caused by a single organism [1,2].

In Indonesia, epidemiological data on bacterial vaginosis (BV) are limited, and most available evidence is derived from small, region-specific studies. These reports suggest prevalence estimates ranging from 10% to 25% among women of reproductive age, with potentially higher rates in selected high-risk populations [6]. Globally, BV remains the most prevalent cause of vaginal discharge among women of reproductive age, with reported prevalence rates ranging from 23% to 29% [6,7]. The burden is notably higher among women with specific risk factors such as high sexual activity, frequent vaginal douching, or recurrent vaginal infections [6,7].

The clinical significance of BV extends well beyond symptomatic discomfort. BV has been consistently associated with an increased risk of acquiring sexually transmitted infections (STIs), including *Chlamydia trachomatis* and human immunodeficiency virus (HIV) [7,8]. Furthermore, BV has been linked to adverse pregnancy outcomes such as preterm labor, premature rupture of membranes, and low birth weight infants [8]. Beyond its physiological effects, BV can also contribute to psychosocial distress, including diminished self-esteem and impaired sexual relationships due to malodorous and excessive vaginal discharge [9]. Economically, BV imposes a substantial burden on healthcare systems through increased clinic visits, recurrent treatments, and long-term reproductive health complications [10].

Diagnosis of BV traditionally relies on Amsel’s clinical criteria and Nugent scoring based on Gram-stained vaginal smears. While these methods are widely used, they are constrained by subjectivity, the need for trained personnel, and delays in result availability [11,12]. While current international guidelines do not recommend routine universal screening for bacterial vaginosis in asymptomatic pregnant women, BV remains clinically relevant in specific contexts. In low-resource and high-prevalence settings, BV has been consistently associated with adverse pregnancy outcomes such as preterm birth and premature rupture of membranes (PPROM), and point-of-care diagnostic tools may offer pragmatic value when laboratory-based testing is unavailable. In response to these limitations, novel diagnostic approaches have emerged—most notably rapid enzymatic tests that promise enhanced accessibility and efficiency.

One such innovation is the BV Rapid Blue test, an enzymatic assay designed to detect sialidase activity—a biomarker produced by BV-associated bacteria—directly from vaginal discharge samples. This point-of-care diagnostic tool delivers results in under 15 minutes, does not require complex laboratory infrastructure, and can be administered in primary healthcare settings [13,14]. International studies have demonstrated that BV Rapid Blue offers excellent diagnostic performance, with reported sensitivities ranging from 88% to 100% and specificities from 95% to 97.8% when compared with the Nugent scoring system as the gold standard [15]. In addition to its diagnostic accuracy, BV Rapid Blue is also considered a cost-effective alternative to molecular methods such as polymerase chain reaction (PCR), making it particularly appealing for use in low-resource settings [13–15].

Despite promising global evidence, no local data are currently available regarding the diagnostic accuracy of BV Rapid Blue or the clinical profiles of pregnant women with BV in Indonesia, specifically at the Dr. Slamet Regional General Hospital (RSUD Dr. Slamet Garut). Therefore, this study aimed to evaluate the sensitivity and specificity of BV Rapid Blue compared to Amsel’s criteria, to describe the characteristics of BV among pregnant patients in this setting, and to analyze the correlation between clinical presentation and diagnostic findings. The results are expected to provide robust scientific evidence supporting the integration of BV Rapid Blue into routine diagnostic protocols, thereby enhancing reproductive health services and facilitating early, accurate, and accessible detection of BV in the Indonesian healthcare system.

## Materials and methods

This study was approved by the Institutional Review Board under approval number DP.04.03/D.XIV.6.5/148/2025.

### Study design and methodology

This cross-sectional diagnostic accuracy study was conducted from April to May 2025 to compare the performance of the BV Rapid Blue enzymatic assay with Amsel’s clinical criteria in diagnosing bacterial vaginosis (BV) among pregnant women. Amsel’s criteria were used as a pragmatic clinical reference standard in this study because Nugent scoring, although internationally recognized as the laboratory gold standard for bacterial vaginosis diagnosis, was not routinely available and was impractical to implement in the study setting. Therefore, the diagnostic performance of BV Rapid Blue was evaluated against Amsel’s criteria within this real-world, resource-limited clinical context.

Sample size estimation was performed based on diagnostic accuracy methodology for sensitivity assessment, following established approaches described by Buderer (1996) and Flahault et al. (2005) [16,17]. A two-sided significance level (α = 0.05; Zα = 1.96) and a statistical power of 80% (Zβ = 0.84) were applied to ensure methodological rigor and avoid directional bias. The calculation assumed an expected sensitivity of 95% for the BV Rapid Blue test, based on prior validation studies, with a minimum acceptable sensitivity of 80%. Based on these parameters, the minimum number of BV-positive cases required was 42. Assuming an estimated BV prevalence of approximately 32% among pregnant women, the total required sample size was calculated to be at least 131 participants to ensure an adequate number of BV-positive cases for reliable sensitivity estimation. Participants were recruited using a consecutive sampling approach, whereby all eligible pregnant women were enrolled in the order of presentation until the required sample size was achieved.

### Study eligibility

The study population comprised pregnant women in the third trimester who presented to the Emergency Department (ED) or the Obstetrics and Gynecology Outpatient Clinic at RSUD Dr. Slamet Garut during the study period and met the inclusion criteria without fulfilling any exclusion criteria.

Inclusion Criteria:

- Aged 18 years or older and in the third trimester
- History of vaginal intercourse within the past three months
- Willing to undergo pelvic examination
- Vaginal pH > 4.5
- Provided informed consent to participate

Exclusion Criteria:

- Pregnancies with fetal congenital anomalies
- Maternal undernutrition, defined as underweight body mass index Fetal growth restriction (FGR)
- Antepartum hemorrhage due to placenta previa
- Multiple gestations
- Pre-existing maternal comorbidities that could affect pregnancy outcomes or vaginal infection status
- Maternal fever suggestive of chorioamnionitis supported by laboratory findings
- Prior diagnosis of infectious vaginitis or cervicitis

### Study instruments and data collection procedures

Eligible pregnant participants who presented to the Emergency Department (ED) or the Obstetrics and Gynecology outpatient clinic were invited to take part in the study. Following a detailed explanation of the study protocol, written informed consent was obtained from those who agreed to participate. Each participant was then asked to complete a structured questionnaire capturing relevant sociodemographic and clinical information. Subsequently, two vaginal swab specimens were collected: the first was used to assess vaginal pH and evaluate Amsel’s clinical criteria for bacterial vaginosis, while the second was utilized for the BV Rapid Blue enzymatic diagnostic test.

Participants who met the study criteria were examined in the dorsal lithotomy position under proper illumination using a sterile speculum. Two sterile vaginal swabs were collected from the posterior fornix, ensuring minimal contact with the vaginal walls during insertion or withdrawal. According to Amsel’s Criteria, diagnosis was based on clinical findings and microscopic evaluation of a wet mount prepared from the vaginal discharge. A positive diagnosis required at least three of the following four criteria:

1. Homogeneous, thin, grayish-white discharge uniformly coating the vaginal walls
2. Presence of ≥ 20% clue cells on wet mount microscopy
3. Vaginal fluid pH > 4.5 measured with pH indicator paper
4. Positive whiff test (fishy odor upon addition of 10% KOH)

The BV Rapid Blue test is an enzymatic colorimetric assay designed to detect the presence of sialidase activity, an enzyme produced by pathogenic bacteria commonly associated with bacterial vaginosis. Patient eligibility was confirmed before testing, including confirmation of no recent antibiotic use or vaginal douching that could interfere with test results. A sterile swab is used to collect vaginal discharge from the posterior vaginal wall, avoiding contact with the vaginal mucosa. The collected specimen is then applied to the test device, and a specific reagent is added following the manufacturer’s (Mingwood Biotechnology, Ltd.) instructions. The mixture is left to react for 10–15 minutes, allowing the enzymatic process to occur. A positive result is indicated by a distinct blue color change on the test area, reflecting the presence of sialidase and, consequently, a presumptive diagnosis of bacterial vaginosis.

To minimize diagnostic bias, the evaluators performing the BV Rapid Blue test were blinded to the results of Amsel’s criteria assessment. However, complete blinding was not feasible for the clinician assessing Amsel’s criteria, as this evaluation was performed during routine clinical examination. This limitation has been transparently acknowledged.

### Statistical analysis

Data were analyzed using both descriptive and analytical statistical methods. Categorical variables were summarized as frequencies and percentages, while continuous variables were reported using means and standard deviations for normally distributed data, and medians with ranges for skewed distributions. The Kolmogorov-Smirnov test was employed to assess the normality of continuous variables. Diagnostic performance was evaluated using 2 x 2 contingency tables to calculate sensitivity, specificity, and overall diagnostic accuracy. Comparative analyses of diagnostic test performance were conducted using Chi-square tests for proportions. For continuous variables, independent t-tests were used for normally distributed data, and the Mann-Whitney U test for non-normally distributed data. To explore factors associated with BV diagnosis within this cross-sectional diagnostic accuracy study, multivariate logistic regression analysis was performed. All statistical analyses were conducted using IBM SPSS 22.0 and GraphPad Prism 10.0. A two-sided p-value of <0.05 was considered statistically significant.

## Results

**Fig 1:**
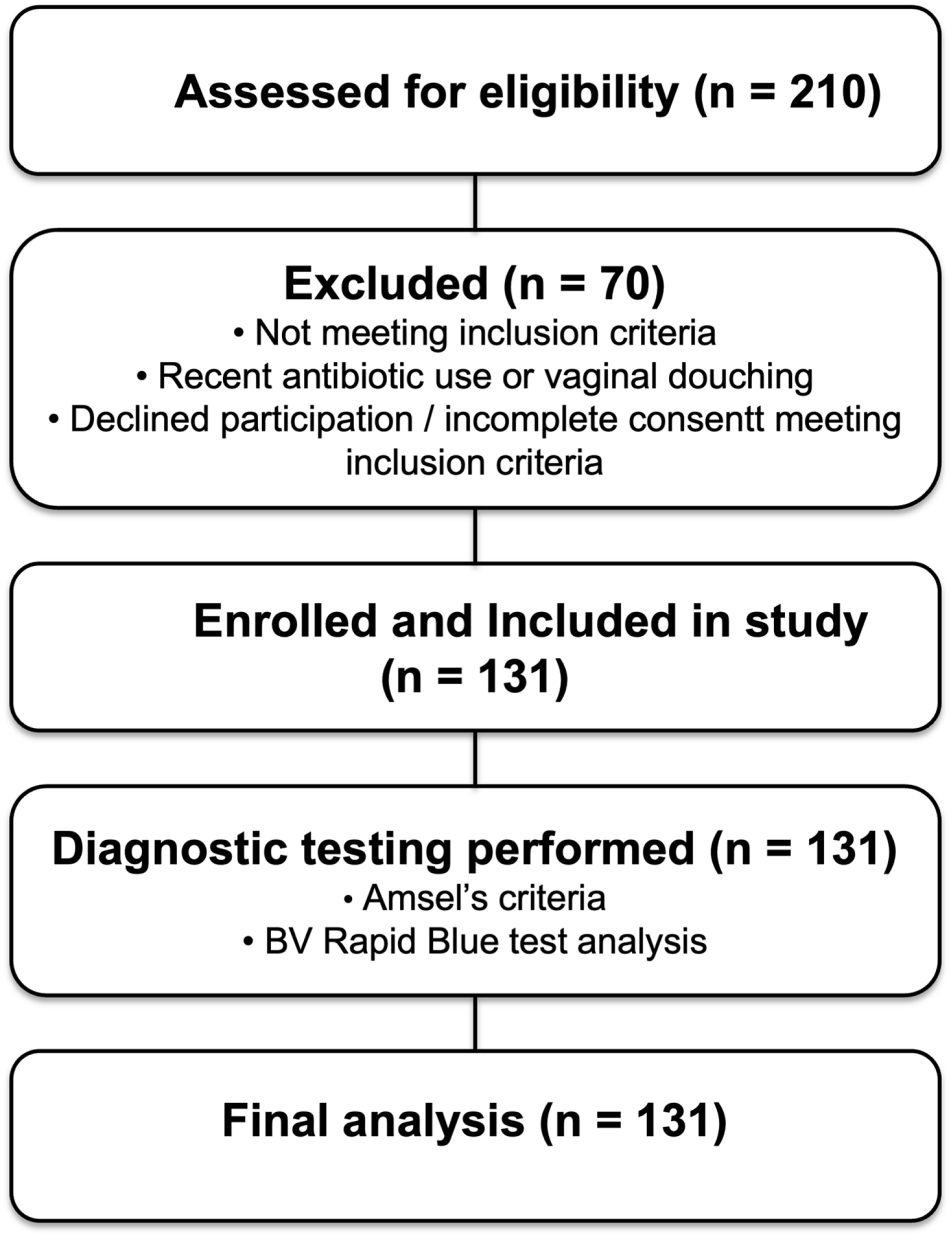
Participant Flow Diagram.

A total of 131 pregnant women in the third trimester participated in this study. A flowchart illustrating participant recruitment, eligibility assessment, exclusion, and inclusion in accordance with STARD 2015 guidelines [18], is presented in Fig 1. Of 210 pregnant women assessed for eligibility, 131 met the inclusion criteria and were enrolled for diagnostic assessment using Amsel’s criteria and BV Rapid Blue testing (Fig 1).

All participants met the eligibility criteria and were enrolled from the emergency department (ED) or the obstetrics and gynecology clinic. All subjects were pregnant women in their third trimester. Each subject provided informed consent and completed a structured questionnaire. Vaginal samples were collected for both Amsel’s criteria assessment and BV Rapid Blue testing to evaluate the diagnostic performance and associated maternal characteristics.

Continuous variables were assessed for normality. As maternal age and gestational age showed non-normal distributions, they are presented as median with interquartile range (IQR), along with minimum and maximum values. The study enrolled 131 pregnant women with a median age of 28.2 years (range: 18–44). Most participants were aged 25–35 years (53.4%). The majority had completed senior high school (45.8%), and over half were multigravida (56.5%). The median gestational age at examination was 38.2 weeks (Table 1).

**Table 1:** Baseline Characteristics of Study Participants (n = 131).

Characteristic	Frequency (%) or Value
**Maternal Age (years)**	
18–24	43 (32.8%)
25–35	70 (53.4%)
>35	18 (13.8%)
Median (IQR; Min–Max)	28.2 (23.5–32.0; 18.0–44.0)
**Education Level**	
Elementary (SD)	18 (13.8%)
Junior High (SMP)	31 (23.6%)
Senior High (SMA)	60 (45.8%)
Higher Education (D3/S1)	22 (16.8%)
**Gravida**	
Primigravida	57 (43.5%)
Multigravida	74 (56.5%)
**Gestational Age (weeks)**	
Median (IQR; Min–Max)	38.2 (37-40; 32.0–42.0)

*Notes: Data were analyzed using both descriptive and analytical statistical methods.

All participants reported vaginal discharge. Nearly half had a history of severe preeclampsia (46.6%), while 32.1% experienced premature rupture of membranes. BV recurrence was reported in 56.5% of cases, and 15.3% showed evidence of secondary infection. No cases of chorioamnionitis were documented (Table 2).

**Table 2:** Maternal Clinical Characteristics (n = 131).

Clinical Characteristic	n (%)
**History of Vaginal Discharge**	
Yes	131 (100%)
No	0 (0%)
**History of STIs**	
Yes	4 (3.1%)
No	127 (96.9%)
**Severe Preeclampsia**	
Yes	61 (46.6%)
No	70 (53.4%)
**Premature Rupture of Membranes**	
Yes	42 (32.1%)
No	89 (67.9%)
**Fetal Distress**	
Yes	11 (8.4%)
No	120 (91.6%)
**Threatened Preterm Labor**	
Yes	12 (9.1%)
No	119 (90.9%)
**Recurrence o**f BV	
Yes	74 (56.5%)
No	57 (43.5%)
**Secondary Infection**	
Yes	20 (15.3%)
No	111 (84.7%)
**Chorioamnionitis**	
Yes	0 (0%)
No	131 (100%)

*Notes: Data were analyzed using both descriptive and analytical statistical methods.

In the present study, 80 out of 131 pregnant women (61.1%) were diagnosed with bacterial vaginosis (BV) using the BV Rapid Blue test, indicating a relatively high prevalence. The BV Rapid Blue test demonstrated strong agreement with Amsel’s criteria (p < 0.0001). Diagnostic performance analysis showed a sensitivity of 95.4% (95% CI: 84.5–99.4) and a specificity of 55.7% (95% CI: 44.7–66.2). The positive predictive value (PPV) was 51.3% (95% CI: 40.0–62.5), while the negative predictive value (NPV) was 96.1% (95% CI: 86.5–99.5). These findings indicate that the BV Rapid Blue test has high sensitivity and excellent NPV, supporting its utility as a reliable screening tool for ruling out BV when the test result is negative (Table 3).

**Table 3:** Diagnostic Test Results of the BV Rapid Blue Examination Compared to Amsel’s Criteria.

BV Rapid Blue (Index Test)	Amsel (+)	Amsel (−)	Total
**Positive**	41 (TP)	39 (FP)	80
**Negative**	2 (FN)	49 (TN)	51
**Total**	43	88	131

Notes: *) Chi-Square Test; Diagnostic performance (95% CI): Sensitivity: 95.4% (84.5–99.4); Specificity: 55.7% (44.7–66.2); PPV: 51.3% (40.0–62.5); NPV: 96.1% (86.5–99.5).

*TP = True Positive; *FP = False Positive; *FN = False Negative; *TN = True Negative; *PPV: Positive Predictive Value; *NPV: Negative Predictive Value

To identify independent predictors of bacterial vaginosis (BV), a multivariate logistic regression analysis was conducted, adjusting for relevant covariates. The model demonstrated that a history of BV recurrence and presence of secondary infection were significantly associated with increased odds of BV diagnosis. Conversely, higher educational attainment appeared to be a protective factor. The detailed results are presented in Table 4.

**Table 4:** Multivariate Logistic Regression.

Predictor Variable	B	SE	Odds Ratio (OR)	95% Confidence Interval (CI)	p-value
BV Recurrence	1.455	0.312	4.29	2.35–7.83	< 0.0001
Secondary Infection	0.940	0.365	2.56	1.23–5.31	0.010
Higher Education Level (S1/D3)	−0.653	0.281	0.52	0.30–0.91	0.020

*Notes: **B** = regression coefficient; SE = standard error; OR = odds ratio; CI = confidence interval; **p** = probability value. A p-value of < 0.05 was considered statistically significant.

Multivariate logistic regression revealed BV recurrence (OR = 4.29, p < 0.0001) and secondary infection (OR = 2.56, p = 0.01) as significant predictors of BV, while higher education (S1/D3) was protective (OR = 0.52, p = 0.02).

## Discussion

This cross-sectional diagnostic accuracy study evaluated the performance of the BV Rapid Blue test using Amsel’s criteria as the pragmatic clinical reference standard and described maternal characteristics among the study population. Most participants were aged 25–35 years, multigravida, and had completed secondary-level education. The universal presence of vaginal discharge suggests that the cohort represented a symptomatic pregnant population, although only a small proportion of participants reported a history of sexually transmitted infections (STIs).

The prevalence of BV observed in this study was higher than that reported in several previous Indonesian studies, which have demonstrated marked regional variation [19–21]. This finding may reflect differences in study populations, local risk profiles, and healthcare access, underscoring the importance of context-specific epidemiological data when interpreting BV burden during pregnancy.

BV Rapid Blue demonstrated strong agreement with Amsel’s criteria and showed excellent sensitivity and negative predictive value, indicating its reliability as a rule-out test for BV. These findings are consistent with international studies reporting favorable diagnostic performance of sialidase-based point-of-care assays when compared with Nugent scoring [17,22,23]. However, the moderate specificity (55.7%) and positive predictive value observed in this study limit the use of BV Rapid Blue as a stand-alone diagnostic test because false-positive results may occur. Therefore, positive test results should be interpreted together with clinical findings or confirmed using additional diagnostic methods whenever available. From a pathophysiological perspective, the diagnostic performance of BV Rapid Blue is supported by the role of sialidase in BV-associated dysbiosis. Sialidase-producing bacteria contribute to mucosal degradation, immune evasion, and biofilm formation on the vaginal epithelium, facilitating persistent infection and recurrence [24–26]. This mechanism provides a plausible biological explanation for the observed findings, particularly the higher odds of BV diagnosis among women with recurrent BV and secondary infection. Conversely, higher educational attainment appeared to confer a protective effect, aligning with previous evidence linking education level to health literacy and preventive behaviors [27,28].

Premature rupture of membranes and preeclampsia were observed among participants in this study. Previous studies have reported associations between BV and adverse pregnancy outcomes, including premature rupture of membranes and preterm birth [28, 29]. However, the present study was not designed to evaluate causal relationships or adjusted associations between BV status and obstetric outcomes. Therefore, these findings should be interpreted descriptively and as hypothesis-generating rather than evidence of a causal relationship.

Despite its favorable diagnostic performance, the clinical scope of BV Rapid Blue must be interpreted cautiously. As a sialidase-based assay, it is designed to detect BV-associated anaerobic activity and does not identify other causes of vaginitis, such as vulvovaginal candidiasis or Group B Streptococcus colonization. Accordingly, BV Rapid Blue should be regarded as a targeted diagnostic tool for BV rather than a comprehensive test for all vaginal infections.

Importantly, this study does not advocate routine universal BV screening during pregnancy, which is not supported by current international guidelines. Instead, the findings should be interpreted within the context of high-prevalence, resource-limited settings where laboratory-based diagnostics such as Nugent scoring may not be readily available. In such environments, rapid point-of-care tests may offer pragmatic value for clinical decision-making, particularly for excluding BV and facilitating timely management.

Overall, this study is exploratory in nature and aims to provide preliminary evidence on the feasibility and diagnostic performance of BV Rapid Blue in routine antenatal care settings. The findings highlight the potential role of accessible point-of-care diagnostics in improving early detection and management of BV in pregnancy, particularly in settings with limited laboratory infrastructure.

## Limitations

This study has several limitations that should be considered when interpreting the findings. First, BV Rapid Blue is a sialidase-based assay and therefore does not detect non–BV-related vaginal infections such as candidiasis or Group B Streptococcus colonization. As a result, it cannot be used as a comprehensive diagnostic tool for all causes of vaginitis. Second, Amsel’s criteria were used as the pragmatic clinical reference standard instead of Nugent scoring, which is internationally recognized as the laboratory reference standard for BV diagnosis. The use of a clinical reference standard may introduce misclassification bias and potentially affect estimates of diagnostic accuracy.

Additionally, the study was conducted in a single-center, high-prevalence population, which may limit generalizability to lower-risk settings. Finally, the study was designed to evaluate diagnostic performance in a real-world, resource-limited context and was not intended to assess the impact of BV testing on pregnancy outcomes or to support routine screening recommendations.

## Conclusions

In conclusion, the BV Rapid Blue test demonstrated high sensitivity and negative predictive value when evaluated against Amsel’s criteria as the pragmatic clinical reference standard, supporting its potential role as a rapid screening tool for ruling out bacterial vaginosis in pregnant women. However, its moderate specificity indicates that positive results should be interpreted alongside clinical findings or confirmed using additional diagnostic methods when available. These findings suggest that BV Rapid Blue may have pragmatic value in real-world clinical settings where laboratory-based diagnostics are limited. Further longitudinal and interventional studies are needed to determine whether the use of rapid point-of-care BV testing can improve maternal or neonatal outcomes.

## Supporting information

S1 Dataset Anonymized minimal dataset used for the analyses in this study. (XLSX)
