# Supplementary material for: Association between bacterial vaginosis prevalence and maternal characteristics in pregnant women: Diagnostic comparison of BV Rapid Blue and Amsel’s criteria
Source: PLoS One. 2026 Aug 14;21(8):e0355948. doi: 10.1371/journal.pone.0355948 (PMC13475853; doi:10.1371/journal.pone.0355948)
Supplement: Supplementary file 1 [file pone.0355948.s003.docx]

**Response to Editor and Reviewers**

Manuscript ID: **PONE-D-25-34601**
Title: *Association Between Bacterial Vaginosis Prevalence and Maternal Characteristics in Pregnant Women: Diagnostic Comparison of BV Rapid Blue and Amsel’s Criteria*

We thank the Academic Editor and both reviewers for their detailed and constructive comments. We have carefully addressed each point raised and revised the manuscript accordingly. Below, we provide a point-by-point response. All changes are highlighted in the revised manuscript with tracked changes.

## **Reviewer #1**

### **Comment 1**

Rapid Blue is only capable of detecting sialidase… There is no possibility of detecting other vaginal conditions such as candida or streptococcus… A comment on that would be appreciated.

**Response:**
We agree with the reviewer. BV Rapid Blue detects elevated vaginal sialidase activity, which is specific to BV-associated anaerobic bacteria and does not identify other vaginal infections such as vulvovaginal candidiasis or Group B Streptococcus. In contrast, fresh wet mount microscopy used as part of Amsel’s criteria may allow visualization of alternative vaginal conditions.

**Changes made:**
This limitation has been explicitly acknowledged in the **Discussion** and further emphasized in a newly added **Limitations** section. We clarified that BV Rapid Blue is intended as a targeted point-of-care test for BV rather than a comprehensive diagnostic tool for all causes of vaginitis.

### **Comment 2**

Pregnancy guidelines do not support BV testing in pregnancy. So why should we check?

**Response:**
We appreciate this important point. While current international guidelines do not recommend routine universal BV screening in asymptomatic pregnant women, BV remains clinically relevant in certain contexts. In low-resource and high-prevalence settings, BV is associated with adverse pregnancy outcomes such as preterm birth and PPROM, and point-of-care diagnostics may offer pragmatic value when laboratory-based testing is unavailable.

**Changes made:**
We have added a clarifying paragraph in both the **Introduction** and **Discussion** explaining the contextual rationale for BV assessment in pregnancy, emphasizing local epidemiology, resource-limited settings, and the exploratory nature of evaluating alternative diagnostic tools rather than advocating routine screening.

## **Reviewer #2**

### **Comment 1**

The title is grammatically awkward.

**Response:**
We agree.

**Changes made:**
The title has been revised to:
**“Association Between Bacterial Vaginosis Prevalence and Maternal Characteristics in Pregnant Women: Diagnostic Comparison of BV Rapid Blue and Amsel’s Criteria.”**

### **Comment 2**

The Abstract repeats entire sentences and should be shortened.

**Response:**
We agree that the Abstract required revision.

**Changes made:**
The Abstract has been fully rewritten, shortened to within 150–250 words, and redundancy with the Introduction and Results has been removed. The revised Abstract now presents a concise summary without verbatim repetition.

### **Comment 3**

References 12–13 are not relevant to BV diagnostics.

**Response:**
We thank the reviewer for identifying these discrepancies.

**Changes made:**
References 12 and 13 have been removed. They were replaced with more relevant literature focusing on BV pathogenesis and diagnostic performance. Additionally, a more recent validation study supporting BV Rapid Blue was added to strengthen the evidence base.

### **Comment 4**

Indonesian prevalence data should be introduced earlier.

**Response:**
We agree.

**Changes made:**
Regional Indonesian prevalence studies are now cited earlier in the **Introduction** to provide clearer epidemiological context.

### **Comment 5**

Amsel’s criteria are not an ideal gold standard; Nugent scoring is preferred.

**Response:**
We fully acknowledge this limitation. Nugent scoring is internationally recognized as the laboratory reference standard. However, due to limited laboratory infrastructure in the study setting, Amsel’s criteria were used as a pragmatic reference standard.

**Changes made:**
The **Methods** section now explicitly describes the study as a **cross-sectional diagnostic accuracy study**. Justification for using Amsel’s criteria is provided, and the potential for misclassification bias and inferior sensitivity/specificity compared with Nugent scoring is clearly acknowledged in the **Limitations** section.

### **Comment 6**

Blinding is missing.

**Response:**
We agree that blinding is an important aspect of diagnostic accuracy studies.

**Changes made:**
The **Methods** section now specifies whether evaluators of BV Rapid Blue and Amsel’s criteria were blinded to each other’s results. Where blinding was not feasible, this has been transparently acknowledged as a limitation.

### **Comment 7**

Consecutive enrollment should be supported with a flow chart; STARD 2015 is recommended.

**Response:**
We thank the reviewer for this recommendation.

**Changes made:**
A participant flow diagram has been added to illustrate recruitment and inclusion. Reporting was revised to better align with **STARD 2015 guidelines**, which are now cited.

### **Comment 8**

Inconsistent data-collection periods.

**Response:**
We apologize for this inconsistency.

**Changes made:**
The data-collection period has been corrected and harmonized throughout the Abstract and Methods sections.

### **Comment 9**

Sample size calculation combines one-sided and two-sided tests.

**Response:**
We agree clarification was required.

**Changes made:**
The sample size calculation has been revised and is now explicitly based on established diagnostic accuracy methodology, with appropriate citation.

### **Comment 10**

Continuous variables lack SDs; trimester restriction not justified.

**Resposne:**
Continuous variables were assessed for normality. As maternal age and gestational age showed non-normal distributions, they are presented as median with interquartile range (IQR), along with minimum and maximum values.

Pregnant women aged ≥18 years, at three trimesters, were eligible for inclusion are clearly stated in the inclusion criteria (methods) and result sections.

### **Comment 11**

GraphPad Prism cannot run multivariate logistic regression.

**Response:**
We thank the reviewer for noting this.

**Changes made:**
The **Statistical Analysis** section has been revised to specify the additional software (IBM SPSS 22.0) used for analyses beyond GraphPad Prism.

### **Comment 12**

Tables contain inconsistencies and lack CIs and significance testing.

**Response:**
We appreciate the reviewer’s careful review of the tables.

**Changes made:**
All tables have been corrected, totals recalculated, decimal formatting standardized, and discrepancies resolved. Statistical comparisons between BV-positive and BV-negative groups have been added, along with **95% confidence intervals** for sensitivity and specificity.

### **Comment 13**

Tables are not cited in the text and table notes are redundant.

**Response:**
We apologize for this oversight.

**Changes made:**
All tables (Tables 1–4) are now appropriately cited in the Results section. Redundant methodological statements have been removed from table notes.

### **Comment 14**

Discussion is overly long and repetitive.

**Response:**
We agree.

**Changes made:**
The Discussion has been substantially condensed and reorganized into concise, interpretive paragraphs, eliminating repeated numerical results and improving logical flow.

### **Comment 15**

References require extensive revision.

**Response:**
We agree.

**Changes made:**
The reference list has been fully revised to ensure relevance, consistent Vancouver formatting, corrected numbering, and complete in-text citation matching.

### **Comment 16**

No Limitations section.

**Response:**
We agree this is mandatory.

**Changes made:**
A dedicated **Limitations** section has been added, addressing diagnostic reference standards, blinding, study setting, and generalizability.
