# Supplementary material for: Association between bacterial vaginosis prevalence and maternal characteristics in pregnant women: Diagnostic comparison of BV Rapid Blue and Amsel’s criteria
Source: PLoS One. 2026 Aug 14;21(8):e0355948. doi: 10.1371/journal.pone.0355948 (PMC13475853; doi:10.1371/journal.pone.0355948)
Supplement: Supplementary file 2 [file pone.0355948.s004.docx]

PONE-D-25-34601R2
Association Between Bacterial Vaginosis Prevalence and Maternal Characteristics in Pregnant Women: Diagnostic Comparison of BV Rapid Blue and Amsel’s Criteria
Dr. Muhammad Alamsyah Aziz

Dear Dr. Aziz,

We've checked your submission and before we can proceed, we need you to address the following issues:

1. Please upload a new copy of Figure 1 as the detail is not clear. Please follow the link for more information: <https://journals.plos.org/plosone/s/figures>

Response:

We have uploaded a revised version of Figure 1 with improved resolution (300 dpi), enhanced text clarity, and optimized formatting in accordance with PLOS ONE figure preparation guidelines. The figure has been recreated to ensure readability and publication-quality standards.

2. We note that there were multiple versions of your Manuscript in your submission's file inventory. We have removed the older file(s), retaining the most recent version(s) for editorial review. Please double check your submission file inventory and let us know if any files appear to be outdated or absent.

Response: We have reviewed the submission file inventory and confirm that all current files are up to date and complete. No outdated or missing files were identified.
