# Supplementary material for: Association between bacterial vaginosis prevalence and maternal characteristics in pregnant women: Diagnostic comparison of BV Rapid Blue and Amsel’s criteria
Source: PLoS One. 2026 Aug 14;21(8):e0355948. doi: 10.1371/journal.pone.0355948 (PMC13475853; doi:10.1371/journal.pone.0355948)
Supplement: Supplementary file 3 [file pone.0355948.s005.docx]

Dear Dr. Aziz,

We've checked your submission and before we can proceed, we need you to address the following issues:

1. Thank you for including your ethics statement on the online submission form:  

"This study was approved by the Institutional Review Board under approval number DP.04.03/D.XIV.6.5/148/2025.".

To help ensure that the wording of your manuscript is suitable for publication, would you please also add this statement at the beginning of the Methods section of your manuscript file.

Response:

Dear Editorial Team,

Thank you for your message.

We have added the ethics statement at the beginning of the Methods section of the manuscript file, as requested:

“This study was approved by the Institutional Review Board under approval number DP.04.03/D.XIV.6.5/148/2025.”

Please let us know if any further revisions are required.

Kind regards,
Dr. Aziz
