# Supplementary material for: Association between bacterial vaginosis prevalence and maternal characteristics in pregnant women: Diagnostic comparison of BV Rapid Blue and Amsel’s criteria
Source: PLoS One. 2026 Aug 14;21(8):e0355948. doi: 10.1371/journal.pone.0355948 (PMC13475853; doi:10.1371/journal.pone.0355948)
Supplement: Supplementary file 4 [file pone.0355948.s006.docx]

**Response to Reviewer #2**

We sincerely thank the reviewer for the careful evaluation of our revised manuscript and for the encouraging comments regarding the substantial improvements in internal consistency, language quality, methodological transparency, and interpretation of the findings. We greatly appreciate the reviewer's constructive suggestions, which have further strengthened the manuscript. All comments have been carefully addressed as detailed below.

**Comment 1:**
*The study should be described consistently as a cross-sectional diagnostic accuracy study. Likewise, referring to Amsel's criteria as the "reference standard" may still be misleading, as Nugent scoring is the accepted laboratory reference standard. Describing Amsel's criteria as the pragmatic clinical reference standard would more accurately reflect the study design and setting.*

**Response:**
Thank you for this important suggestion. We have revised the manuscript throughout to consistently describe the study as a **cross-sectional diagnostic accuracy study**. In addition, we have replaced the term "reference standard" with **"pragmatic clinical reference standard"** whenever Amsel's criteria are mentioned. We also explicitly clarified that Nugent scoring remains the internationally accepted laboratory gold standard but was not routinely available in our resource-limited clinical setting. These revisions improve methodological precision and better reflect the study design.

**Comment 2:**
*The discussion of obstetric conditions, particularly preeclampsia and premature rupture of membranes, should remain strictly descriptive. Given the cross-sectional design and the absence of comparative or adjusted analyses, the present study cannot establish associations between BV and these obstetric outcomes.*

**Response:**
We appreciate this valuable comment and fully agree. The Discussion has been revised to ensure that obstetric conditions such as preeclampsia and premature rupture of membranes are presented **descriptively only**. Any wording that could imply causal or independent associations has been removed. We now explicitly state that the present cross-sectional study was not designed to evaluate causal relationships or adjusted associations between bacterial vaginosis and obstetric outcomes, and therefore these findings should be interpreted only as descriptive and hypothesis-generating.

**Comment 3:**
*Although BV Rapid Blue demonstrated excellent sensitivity and negative predictive value, its relatively low specificity (55.7%) limits its performance as a stand-alone diagnostic test because of the potential for false-positive results. The conclusions should therefore emphasize its potential role as a screening or rule-out test.*

**Response:**
Thank you for this insightful recommendation. We have revised both the Discussion and the Conclusions to better emphasize the clinical implications of our diagnostic performance results. The manuscript now clearly states that the **high sensitivity and excellent negative predictive value support the use of BV Rapid Blue primarily as a screening or rule-out test**, particularly in resource-limited settings. We also explicitly acknowledge that its moderate specificity may result in false-positive findings and that positive results should be interpreted alongside clinical assessment or confirmed using additional diagnostic methods whenever available.

**Comment 4:**
*Although the overall quality of the writing has improved considerably, a final careful proofreading is recommended.*

**Response:**
Thank you for this recommendation. The entire manuscript has undergone another comprehensive proofreading to improve grammar, wording, consistency, and overall readability. Minor grammatical inconsistencies and stylistic issues have been corrected throughout the manuscript.

We sincerely thank the reviewer for the thoughtful comments and positive assessment of our work. We believe that these final revisions have substantially improved the clarity, methodological accuracy, and clinical interpretation of the manuscript, and we hope that it is now suitable for publication in *PLOS ONE*.

Sincerely,

Prof. Muhammad Alamsyah Aziz, MD
On behalf of all authors
