# Supplementary material for: Association between bacterial vaginosis prevalence and maternal characteristics in pregnant women: Diagnostic comparison of BV Rapid Blue and Amsel’s criteria
Source: PLoS One. 2026 Aug 14;21(8):e0355948. doi: 10.1371/journal.pone.0355948 (PMC13475853; doi:10.1371/journal.pone.0355948)
Supplement: Peer review history

# Peer review: Decision Letter 0

**DOI:** 10.1371/journal.pone.0355948.r001

**Authors:** Li Zhe

27 Oct 2025

Dear Dr. Aziz,

Thank you for submitting your manuscript to PLOS ONE. After careful consideration, we feel that it has merit but does not fully meet PLOS ONE’s publication criteria as it currently stands. Therefore, we invite you to submit a revised version of the manuscript that addresses the points raised during the review process.

Please submit your revised manuscript by Dec 11 2025 11:59PM. If you will need more time than this to complete your revisions, please reply to this message or contact the journal office at plosone@plos.org. When you're ready to submit your revision, log on to https://www.editorialmanager.com/pone/ and select the 'Submissions Needing Revision' folder to locate your manuscript file.

- A rebuttal letter that responds to each point raised by the academic editor and reviewer(s). You should upload this letter as a separate file labeled 'Response to Reviewers'.
- A marked-up copy of your manuscript that highlights changes made to the original version. You should upload this as a separate file labeled 'Revised Manuscript with Track Changes'.
- An unmarked version of your revised paper without tracked changes. You should upload this as a separate file labeled 'Manuscript'.

If you would like to make changes to your financial disclosure, please include your updated statement in your cover letter. Guidelines for resubmitting your figure files are available below the reviewer comments at the end of this letter.

If applicable, we recommend that you deposit your laboratory protocols in protocols.io to enhance the reproducibility of your results. Protocols.io assigns your protocol its own identifier (DOI) so that it can be cited independently in the future. For instructions see: https://journals.plos.org/plosone/s/submission-guidelines#loc-laboratory-protocols. Additionally, PLOS ONE offers an option for publishing peer-reviewed Lab Protocol articles, which describe protocols hosted on protocols.io. Read more information on sharing protocols at https://plos.org/protocols?utm_medium=editorial-email&utm_source=authorletters&utm_campaign=protocols.

We look forward to receiving your revised manuscript.

Kind regards,

Li Zhe

Academic Editor

PLOS ONE

Journal Requirements:

When submitting your revision, we need you to address these additional requirements.

1.Please ensure that your manuscript meets PLOS ONE's style requirements, including those for file naming. The PLOS ONE style templates can be found at

https://journals.plos.org/plosone/s/file?id=wjVg/PLOSOne_formatting_sample_main_body.pdf and

https://journals.plos.org/plosone/s/file?id=ba62/PLOSOne_formatting_sample_title_authors_affiliations.pdf

2. We note that your Data Availability Statement is currently as follows: “All relevant data are within the manuscript and its Supporting Information files.”

Please confirm at this time whether or not your submission contains all raw data required to replicate the results of your study. Authors must share the “minimal data set” for their submission. PLOS defines the minimal data set to consist of the data required to replicate all study findings reported in the article, as well as related metadata and methods (https://journals.plos.org/plosone/s/data-availability#loc-minimal-data-set-definition).

For example, authors should submit the following data:

- The values behind the means, standard deviations and other measures reported;

- The values used to build graphs;

- The points extracted from images for analysis.

Authors do not need to submit their entire data set if only a portion of the data was used in the reported study.

If your submission does not contain these data, please either upload them as Supporting Information files or deposit them to a stable, public repository and provide us with the relevant URLs, DOIs, or accession numbers. For a list of recommended repositories, please see https://journals.plos.org/plosone/s/recommended-repositories.

If there are ethical or legal restrictions on sharing a de-identified data set, please explain them in detail (e.g., data contain potentially sensitive information, data are owned by a third-party organization, etc.) and who has imposed them (e.g., an ethics committee). Please also provide contact information for a data access committee, ethics committee, or other institutional body to which data requests may be sent. If data are owned by a third party, please indicate how others may request data access.

3. We note you have included a table to which you do not refer in the text of your manuscript. Please ensure that you refer to Table 1, 2 and 3 in your text; if accepted, production will need this reference to link the reader to the Table.

4. If the reviewer comments include a recommendation to cite specific previously published works, please review and evaluate these publications to determine whether they are relevant and should be cited. There is no requirement to cite these works unless the editor has indicated otherwise.

[Note: HTML markup is below. Please do not edit.]

Reviewer's Responses to Questions

**Comments to the Author**

1. Is the manuscript technically sound, and do the data support the conclusions?

Reviewer #1: Yes

Reviewer #2: No

**********

2. Has the statistical analysis been performed appropriately and rigorously?

Reviewer #1: Yes

Reviewer #2: No

**********

3. Have the authors made all data underlying the findings in their manuscript fully available?

The PLOS Data policy

Reviewer #1: Yes

Reviewer #2: No

**********

4. Is the manuscript presented in an intelligible fashion and written in standard English?

Reviewer #1: Yes

Reviewer #2: No

**********

Reviewer #1: Rapid blue is only capable of detecting sialidase, which means BV-associated bacteria. There is no possibilities of detecting other vaginal conditions such as candida or streptococcus that may be relevant in pregnancy (especially the latter). On the other hand fresh mount microscopy which is used for Amsel's criteria is capable of such diagnosis. A comment on that would be appreciated.

Furthermore, pregnancy guidelines do not support BV testing in pregnancy. So why should we check? A comment on that will be appreciated as well.

Reviewer #2: This paper is relevant to the local context and could contribute to implementing more modern diagnostic tests within the public health system, but it requires extensive review. The main points are highlighted here.

The title is grammatically awkward. Suggested revision: “Association Between Bacterial Vaginosis Prevalence and Maternal Characteristics in Pregnant Women: Diagnostic Comparison of BV Rapid Blue and Amsel’s Criteria.”

The Abstract is structured but repeats entire sentences from the Introduction and Results and should be shortened (150–250 words). Also, it should be rewritten so as not to reproduce verbatim what is stated in the article's sections.

In the introduction, a closer look at the reference list shows some discrepancies: References 12–13 are not entirely relevant to BV diagnostics. Ref. 12 (Aitken et al., 2019) addresses cervical intraepithelial neoplasia management, not BV. Ref. 13 (Cortés Bordoy et al., 2023) concerns an HPV-related vaginal gel study and is unrelated to BV pathogenesis or testing. Reference 15, cited as supporting the BV Rapid Blue diagnostic performance, is indeed appropriate (Shujatullah et al., 2010, on OSOM BV Blue), but would benefit from an additional recent validation paper for stronger evidence.

The text mentions prevalence data from Indonesian regions but does not cite local sources until much later (Refs 16–19). It would be preferable to introduce these regional studies earlier in the introduction for smoother context linkage.

In methodology, the study design needs revision. As a suggestion, “A cross-sectional diagnostic accuracy design is indeed the correct approach for evaluating the performance of an index test (BV Rapid Blue) against a reference standard (Amsel’s criteria)”. However, Amsel’s criteria are not an ideal gold standard. Nugent scoring (Gram-stained smear) is internationally recognized as the reference method. Using Amsel instead of Nugent should be justified clearly (e.g., limited lab access, pilot validation). Using Amsel’s criteria (a clinical, not laboratory-based standard) can lead to misclassification bias. The study should at least note that Amsel has inferior sensitivity/specificity compared with Nugent.

The blinding is missing. There is no mention of whether the evaluators of BV Rapid Blue and Amsel’s test were blinded to each other’s results, a significant omission in diagnostic accuracy research.

While consecutive enrollment reduces selection bias, it should be supported with a flow chart (e.g., “165 eligible → 131 included → 0 excluded”). I strongly recommend the STARD 2015 overview as a guideline for reporting diagnostic accuracy studies (Cohen JF, Korevaar DA, Altman DG, et al. STARD 2015 guidelines for reporting diagnostic accuracy studies: explanation and elaboration. BMJ Open. 2016;6(11):e012799. Published 2016 Nov 14. doi:10.1136/bmjopen-2016-012799).

There are inconsistent data-collection periods: the Abstract says “March–April 2025”; the methods say “April–May 2025.” Must be corrected. The sample size calculation: Combines one-sided and two-sided tests without justification. Should cite Buderer, 1996, or Flahault et al., 2005 for diagnostic accuracy sample estimation.

The continuous variables (age, gestational age) were reported only as means and ranges; however, they should include SDs. If all participants were third-trimester pregnancies, the authors must justify excluding participants from other trimesters.

In the statistical analysis, GraphPad Prism 10 cannot run multivariate logistic regression. Please confirm whether a secondary software package (e.g., SPSS, Stata) was used.

In the Results, tables lack proper scientific formatting, with inconsistent decimal places in percentages, and the absence of totals, for instance. Table 3 totals do not add up (41+2+39+49 ≠ 131). The text reports the BV prevalence of 65%, but the table shows 61.1%. Authors should include significance testing (BV+ vs. BV–) for maternal variables. There is a lack of 95% confidence intervals (CIs) for sensitivity and specificity. Repetitions of numerical results in the discussion must be removed. Omitting CIs and failing to discuss prevalence limits are methodological flaws that should be corrected before resubmission.

Another relevant question is the lack of cross-references between the text and the tables. Tables 1–4 are presented, but none are appropriately cited in the text (e.g., “as shown in Table 2”), and are required by PLOS ONE guidelines. The table notes repeat the methodology text (“data analyzed using descriptive and analytical methods”), which is redundant and unnecessary.

The discussion is overly long, overly descriptive, and repetitive, and it copies sections from the Introduction and Results. For instance, “BV Rapid Blue showed strong agreement with Amsel criteria (p < 0.0001)” is repeated three times. “High sensitivity and NPV” appears in consecutive paragraphs. As a suggestion, authors can condense it into 4–5 interpretive paragraphs, with logical transitions (“Our findings suggest…”, “This may explain…”).

The references list requires extensive revision. There are recent and relevant citations (up to 2025), but inconsistent formatting: duplicated numbering (1–5 repeat authors), references 12–14 are unrelated, and the Vancouver style is not standardized. (e.g., periods after authors, DOI without “https://”). Finally, many references in-text citations are missing.

There is no Limitations section, mandatory for observational studies. Many paragraphs of this manuscript require proofreading.

**********

what does this mean?). If published, this will include your full peer review and any attached files.

If you choose “no”, your identity will remain anonymous but your review may still be made public.

**Do you want your identity to be public for this peer review?**  For information about this choice, including consent withdrawal, please see our Privacy Policy

Reviewer #1: No

Reviewer #2: No

**********

[NOTE: If reviewer comments were submitted as an attachment file, they will be attached to this email and accessible via the submission site. Please log into your account, locate the manuscript record, and check for the action link "View Attachments". If this link does not appear, there are no attachment files.]

To ensure your figures meet our technical requirements, please review our figure guidelines: https://journals.plos.org/plosone/s/figures

You may also use PLOS’s free figure tool, NAAS, to help you prepare publication quality figures: https://journals.plos.org/plosone/s/figures#loc-tools-for-figure-preparation.

NAAS will assess whether your figures meet our technical requirements by comparing each figure against our figure specifications.

# Author comment: Author response to Decision Letter 1

**DOI:** 10.1371/journal.pone.0355948.r002

6 Jan 2026

We thank the Academic Editor and both reviewers for their detailed and constructive comments. We have carefully addressed each point raised and revised the manuscript accordingly. Below, we provide a point-by-point response. All changes are highlighted in the revised manuscript with tracked changes.

Reviewer #1

Comment 1

Rapid Blue is only capable of detecting sialidase… There is no possibility of detecting other vaginal conditions such as candida or streptococcus… A comment on that would be appreciated.

Response:

We agree with the reviewer. BV Rapid Blue detects elevated vaginal sialidase activity, which is specific to BV-associated anaerobic bacteria and does not identify other vaginal infections such as vulvovaginal candidiasis or Group B Streptococcus. In contrast, fresh wet mount microscopy used as part of Amsel’s criteria may allow visualization of alternative vaginal conditions.

Changes made:

This limitation has been explicitly acknowledged in the Discussion and further emphasized in a newly added Limitations section. We clarified that BV Rapid Blue is intended as a targeted point-of-care test for BV rather than a comprehensive diagnostic tool for all causes of vaginitis.

Comment 2

Pregnancy guidelines do not support BV testing in pregnancy. So why should we check?

Response:

We appreciate this important point. While current international guidelines do not recommend routine universal BV screening in asymptomatic pregnant women, BV remains clinically relevant in certain contexts. In low-resource and high-prevalence settings, BV is associated with adverse pregnancy outcomes such as preterm birth and PPROM, and point-of-care diagnostics may offer pragmatic value when laboratory-based testing is unavailable.

Changes made:

We have added a clarifying paragraph in both the Introduction and Discussion explaining the contextual rationale for BV assessment in pregnancy, emphasizing local epidemiology, resource-limited settings, and the exploratory nature of evaluating alternative diagnostic tools rather than advocating routine screening.

Reviewer #2

Comment 1

The title is grammatically awkward.

Response:

We agree.

Changes made:

The title has been revised to:

“Association Between Bacterial Vaginosis Prevalence and Maternal Characteristics in Pregnant Women: Diagnostic Comparison of BV Rapid Blue and Amsel’s Criteria.”

Comment 2

The Abstract repeats entire sentences and should be shortened.

Response:

We agree that the Abstract required revision.

Changes made:

The Abstract has been fully rewritten, shortened to within 150–250 words, and redundancy with the Introduction and Results has been removed. The revised Abstract now presents a concise summary without verbatim repetition.

Comment 3

References 12–13 are not relevant to BV diagnostics.

Response:

We thank the reviewer for identifying these discrepancies.

Changes made:

References 12 and 13 have been removed. They were replaced with more relevant literature focusing on BV pathogenesis and diagnostic performance. Additionally, a more recent validation study supporting BV Rapid Blue was added to strengthen the evidence base.

Comment 4

Indonesian prevalence data should be introduced earlier.

Response:

We agree.

Changes made:

Regional Indonesian prevalence studies are now cited earlier in the Introduction to provide clearer epidemiological context.

Comment 5

Amsel’s criteria are not an ideal gold standard; Nugent scoring is preferred.

Response:

We fully acknowledge this limitation. Nugent scoring is internationally recognized as the laboratory reference standard. However, due to limited laboratory infrastructure in the study setting, Amsel’s criteria were used as a pragmatic reference standard.

Changes made:

The Methods section now explicitly describes the study as a cross-sectional diagnostic accuracy study. Justification for using Amsel’s criteria is provided, and the potential for misclassification bias and inferior sensitivity/specificity compared with Nugent scoring is clearly acknowledged in the Limitations section.

Comment 6

Blinding is missing.

Response:

We agree that blinding is an important aspect of diagnostic accuracy studies.

Changes made:

The Methods section now specifies whether evaluators of BV Rapid Blue and Amsel’s criteria were blinded to each other’s results. Where blinding was not feasible, this has been transparently acknowledged as a limitation.

Comment 7

Consecutive enrollment should be supported with a flow chart; STARD 2015 is recommended.

Response:

We thank the reviewer for this recommendation.

Changes made:

A participant flow diagram has been added to illustrate recruitment and inclusion. Reporting was revised to better align with STARD 2015 guidelines, which are now cited.

Comment 8

Inconsistent data-collection periods.

Response:

We apologize for this inconsistency.

Changes made:

The data-collection period has been corrected and harmonized throughout the Abstract and Methods sections.

Comment 9

Sample size calculation combines one-sided and two-sided tests.

Response:

We agree clarification was required.

Changes made:

The sample size calculation has been revised and is now explicitly based on established diagnostic accuracy methodology, with appropriate citation.

Comment 10

Continuous variables lack SDs; trimester restriction not justified.

Resposne:

Continuous variables were assessed for normality. As maternal age and gestational age showed non-normal distributions, they are presented as median with interquartile range (IQR), along with minimum and maximum values.

Pregnant women aged ≥18 years, at three trimesters, were eligible for inclusion are clearly stated in the inclusion criteria (methods) and result sections.

Comment 11

GraphPad Prism cannot run multivariate logistic regression.

Response:

We thank the reviewer for noting this.

Changes made:

The Statistical Analysis section has been revised to specify the additional software (IBM SPSS 22.0) used for analyses beyond GraphPad Prism.

Comment 12

Tables contain inconsistencies and lack CIs and significance testing.

Response:

We appreciate the reviewer’s careful review of the tables.

Changes made:

All tables have been corrected, totals recalculated, decimal formatting standardized, and discrepancies resolved. Statistical comparisons between BV-positive and BV-negative groups have been added, along with 95% confidence intervals for sensitivity and specificity.

Comment 13

Tables are not cited in the text and table notes are redundant.

Response:

We apologize for this oversight.

Changes made:

All tables (Tables 1–4) are now appropriately cited in the Results section. Redundant methodological statements have been removed from table notes.

Comment 14

Discussion is overly long and repetitive.

Response:

We agree.

Changes made:

The Discussion has been substantially condensed and reorganized into concise, interpretive paragraphs, eliminating repeated numerical results and improving logical flow.

Comment 15

References require extensive revision.

Response:

We agree.

Changes made:

The reference list has been fully revised to ensure relevance, consistent Vancouver formatting, corrected numbering, and complete in-text citation matching.

Comment 16

No Limitations section.

Response:

We agree this is mandatory.

Changes made:

A dedicated Limitations section has been added, addressing diagnostic reference standards, blinding, study setting, and generalizability.

Thank You

Attachment Submitted filename: Response to The Editor and Reviewer.docx

# Peer review: Decision Letter 1

**DOI:** 10.1371/journal.pone.0355948.r003

**Authors:** Benjamin Jerry Ridenhour

24 Feb 2026

Dear Dr. Aziz,

Thank you for submitting your manuscript to PLOS ONE. After careful consideration, we feel that it has merit but does not fully meet PLOS ONE’s publication criteria as it currently stands. Therefore, we invite you to submit a revised version of the manuscript that addresses the points raised during the review process.

Please submit your revised manuscript by Apr 10 2026 11:59PM. If you will need more time than this to complete your revisions, please reply to this message or contact the journal office at plosone@plos.org. When you're ready to submit your revision, log on to https://www.editorialmanager.com/pone/ and select the 'Submissions Needing Revision' folder to locate your manuscript file.

- A letter that responds to each point raised by the academic editor and reviewer(s). You should upload this letter as a separate file labeled 'Response to Reviewers'.
- A marked-up copy of your manuscript that highlights changes made to the original version. You should upload this as a separate file labeled 'Revised Manuscript with Track Changes'.
- An unmarked version of your revised paper without tracked changes. You should upload this as a separate file labeled 'Manuscript'.

If applicable, we recommend that you deposit your laboratory protocols in protocols.io to enhance the reproducibility of your results. Protocols.io assigns your protocol its own identifier (DOI) so that it can be cited independently in the future. For instructions see: https://journals.plos.org/plosone/s/submission-guidelines#loc-laboratory-protocols. Additionally, PLOS ONE offers an option for publishing peer-reviewed Lab Protocol articles, which describe protocols hosted on protocols.io. Read more information on sharing protocols at https://plos.org/protocols?utm_medium=editorial-email&utm_source=authorletters&utm_campaign=protocols.

We look forward to receiving your revised manuscript.

Kind regards,

Benjamin Jerry Ridenhour

Academic Editor

PLOS One

Journal Requirements:

If the reviewer comments include a recommendation to cite specific previously published works, please review and evaluate these publications to determine whether they are relevant and should be cited. There is no requirement to cite these works unless the editor has indicated otherwise.

Please review your reference list to ensure that it is complete and correct. If you have cited papers that have been retracted, please include the rationale for doing so in the manuscript text, or remove these references and replace them with relevant current references. Any changes to the reference list should be mentioned in the rebuttal letter that accompanies your revised manuscript. If you need to cite a retracted article, indicate the article’s retracted status in the References list and also include a citation and full reference for the retraction notice.

Additional Editor Comments:

Please see the outstanding concerns of one of the reviewers. I agree that these are issues that need to be addressed prior to acceptance for publication given that they mainly relate to clarity and consistency of the manuscript.

[Note: HTML markup is below. Please do not edit.]

Reviewers' comments:

Reviewer's Responses to Questions

**Comments to the Author**

Reviewer #1: All comments have been addressed

Reviewer #2: (No Response)

**********

2. Is the manuscript technically sound, and do the data support the conclusions?

Reviewer #1: Yes

Reviewer #2: Partly

**********

3. Has the statistical analysis been performed appropriately and rigorously?

Reviewer #1: N/A

Reviewer #2: No

**********

4. Have the authors made all data underlying the findings in their manuscript fully available?

The PLOS Data policy

Reviewer #1: Yes

Reviewer #2: Yes

**********

5. Is the manuscript presented in an intelligible fashion and written in standard English?

Reviewer #1: Yes

Reviewer #2: No

**********

Reviewer #1: all concerns have been addressed and in my opinion the manuscript is acceptable for publication in this journal.

Reviewer #2: The revised manuscript has improved substantially compared with the previous version. The authors have addressed several of the initial methodological and structural concerns, including clarifying the study design as a diagnostic accuracy study, including a participant flow diagram consistent with STARD recommendations, adding a Limitations section, and specifying the statistical software used for multivariate analysis. These revisions strengthen the overall structure and transparency of the manuscript.

However, several important issues remain and should be addressed prior to acceptance.

1. Inconsistency in BV Prevalence Reporting

There is a discrepancy in the reported prevalence of bacterial vaginosis (BV). The Abstract states that BV prevalence was 65%, whereas the contingency table data (Table 3) indicate 80 BV-positive cases out of 131 participants (61.1%).

These values are not equivalent and must be harmonized throughout the manuscript. This inconsistency affects the interpretation of results and should be corrected.

2. Presentation and Formatting of Table 3 (Diagnostic Accuracy Table)

Table 3 remains difficult to interpret. Although the totals sum correctly, the 2×2 diagnostic matrix is not presented in a clear, standard format. For diagnostic accuracy studies, the contingency table should be displayed explicitly (e.g., index test positive/negative versus reference standard positive/negative), including row and column totals.

In its current form, the table lacks clarity and does not fully conform to STARD reporting standards. A conventional 2×2 table format is strongly recommended.

3. Missing 95% Confidence Intervals for Diagnostic Accuracy Parameters

Sensitivity, specificity, PPV, and NPV are reported; however, 95% confidence intervals (CIs) are not provided. The absence of CIs limits interpretability and statistical rigor. STARD guidelines explicitly recommend reporting precision measures for diagnostic accuracy parameters.

The authors should calculate and present 95% CIs for sensitivity and specificity (at minimum), and preferably for PPV and NPV as well.

4. Inconsistency in Inclusion Criteria (Trimester Specification)

There appears to be an inconsistency between versions regarding gestational age eligibility. The Methods section now specifies third-trimester pregnancies, and the Results state that all participants were in the third trimester. However, earlier versions described inclusion regardless of gestational age.

If only third-trimester patients were included, this must be clearly justified in the Methods section, as it impacts external validity and generalizability. If not, the manuscript requires correction for consistency. This is a crucial point.

5. Sample Size Calculation Methodology

The sample size section has been revised; however, the explanation still appears unclear and somewhat contradictory regarding one-sided versus two-sided significance levels. The manuscript should clearly state which approach was ultimately used and cite an established diagnostic accuracy sample size reference (e.g., Buderer, 1996; Flahault et al., 2005). Ambiguity in this section may raise concerns regarding statistical rigor.

Minor questions

a) Grammatical and Language Issues

Several grammatical errors persist and require professional language editing. For example:

• “BV remains prevalence in pregnancy” should read “BV remains prevalent in pregnancy.”

• Minor sentence structure errors appear in the Methods section.

Given the journal’s standards, thorough language revision is recommended.

b) Repetition in the Discussion

Although shortened, the Discussion still repeats key findings (e.g., “strong agreement,” “high sensitivity and NPV”) multiple times. Further condensation into concise interpretive paragraphs would improve readability and scientific tone.

c) Cross-Referencing of Tables

Tables should be consistently cited in the Results section (e.g., “as shown in Table 2”). While improvements have been made, ensure all tables are referenced appropriately in accordance with the journal's formatting guidelines.

Overall Recommendation

The manuscript has improved meaningfully and now presents a clearer diagnostic accuracy framework. However, important methodological clarifications and reporting refinements remain necessary, particularly regarding consistency in prevalence estimates, diagnostic table formatting, and the inclusion of confidence intervals.

With these revisions, the manuscript could be suitable for publication.

**********

what does this mean?). If published, this will include your full peer review and any attached files.

If you choose “no”, your identity will remain anonymous but your review may still be made public.

**Do you want your identity to be public for this peer review?**  For information about this choice, including consent withdrawal, please see our Privacy Policy

Reviewer #1: **Yes:** Lorenzo Agoni

Reviewer #2: No

**********

[NOTE: If reviewer comments were submitted as an attachment file, they will be attached to this email and accessible via the submission site. Please log into your account, locate the manuscript record, and check for the action link "View Attachments". If this link does not appear, there are no attachment files.]

To ensure your figures meet our technical requirements, please review our figure guidelines: https://journals.plos.org/plosone/s/figures

You may also use PLOS’s free figure tool, NAAS, to help you prepare publication quality figures: https://journals.plos.org/plosone/s/figures#loc-tools-for-figure-preparation.

NAAS will assess whether your figures meet our technical requirements by comparing each figure against our figure specifications.

# Author comment: Author response to Decision Letter 2

**DOI:** 10.1371/journal.pone.0355948.r004

15 Apr 2026

Reviewer #1: all concerns have been addressed and in my opinion the manuscript is acceptable for publication in this journal.

Response: We thank the reviewer for the positive evaluation and for considering our manuscript acceptable for publication. We greatly appreciate the time and expertise devoted to reviewing our work.

Reviewer #2: The revised manuscript has improved substantially compared with the previous version. The authors have addressed several of the initial methodological and structural concerns, including clarifying the study design as a diagnostic accuracy study, including a participant flow diagram consistent with STARD recommendations, adding a Limitations section, and specifying the statistical software used for multivariate analysis. These revisions strengthen the overall structure and transparency of the manuscript.

However, several important issues remain and should be addressed prior to acceptance.

1. Inconsistency in BV Prevalence Reporting

There is a discrepancy in the reported prevalence of bacterial vaginosis (BV). The Abstract states that BV prevalence was 65%, whereas the contingency table data (Table 3) indicate 80 BV-positive cases out of 131 participants (61.1%).

These values are not equivalent and must be harmonized throughout the manuscript. This inconsistency affects the interpretation of results and should be corrected.

Response: Thank you for highlighting this discrepancy. We have carefully reviewed the data and corrected the inconsistency. The prevalence of BV has now been harmonized across the Abstract, Results, and Table 3 to reflect the accurate value of 61.1% (80/131 participants). All sections of the manuscript have been updated accordingly.

2. Presentation and Formatting of Table 3 (Diagnostic Accuracy Table)

Table 3 remains difficult to interpret. Although the totals sum correctly, the 2×2 diagnostic matrix is not presented in a clear, standard format. For diagnostic accuracy studies, the contingency table should be displayed explicitly (e.g., index test positive/negative versus reference standard positive/negative), including row and column totals.

In its current form, the table lacks clarity and does not fully conform to STARD reporting standards. A conventional 2×2 table format is strongly recommended.

Response: We appreciate this important suggestion. Table 3 has been revised and reformatted into a standard 2×2 contingency table, clearly presenting:

• Index test (positive/negative)

• Reference standard (positive/negative)

• Row and column totals

This revision aligns with STARD recommendations and improves interpretability.

3. Missing 95% Confidence Intervals for Diagnostic Accuracy Parameters

Sensitivity, specificity, PPV, and NPV are reported; however, 95% confidence intervals (CIs) are not provided. The absence of CIs limits interpretability and statistical rigor. STARD guidelines explicitly recommend reporting precision measures for diagnostic accuracy parameters. The authors should calculate and present 95% CIs for sensitivity and specificity (at minimum), and preferably for PPV and NPV as well.

Response: Thank you for this valuable comment. We have now calculated and included 95% confidence intervals (CIs) for sensitivity and specificity, as well as for PPV and NPV. These have been added to the Results section and corresponding tables to enhance statistical rigor and interpretability.

4. Inconsistency in Inclusion Criteria (Trimester Specification)

There appears to be an inconsistency between versions regarding gestational age eligibility. The Methods section now specifies third-trimester pregnancies, and the Results state that all participants were in the third trimester. However, earlier versions described inclusion regardless of gestational age.

If only third-trimester patients were included, this must be clearly justified in the Methods section, as it impacts external validity and generalizability. If not, the manuscript requires correction for consistency. This is a crucial point.

Response: We appreciate the reviewer’s attention to this critical point. We confirm that only third-trimester pregnancies were included in this study. This has now been:

• Clearly stated and justified in the Methods section, and

• Consistently reflected in the Results section

We have added a brief justification explaining that third-trimester inclusion was chosen to ensure clinical relevance and homogeneity of the study population. We also acknowledge this as a limitation affecting generalizability in the Limitations section.

5. Sample Size Calculation Methodology

The sample size section has been revised; however, the explanation still appears unclear and somewhat contradictory regarding one-sided versus two-sided significance levels. The manuscript should clearly state which approach was ultimately used and cite an established diagnostic accuracy sample size reference (e.g., Buderer, 1996; Flahault et al., 2005). Ambiguity in this section may raise concerns regarding statistical rigor.

Response: Thank you for pointing out the lack of clarity. The sample size calculation section has been revised to:

• Clearly specify the use of a two-sided significance level

• Remove any ambiguous or contradictory statements

• Include appropriate references, specifically Buderer (1996) and Flahault et al. (2005)

This revision ensures methodological transparency and consistency.

Minor questions

a) Grammatical and Language Issues

Several grammatical errors persist and require professional language editing. For example:

• “BV remains prevalence in pregnancy” should read “BV remains prevalent in pregnancy.”

• Minor sentence structure errors appear in the Methods section.

Given the journal’s standards, thorough language revision is recommended.

Response: We appreciate this observation. The manuscript has undergone comprehensive professional language editing, and all identified grammatical issues (including the example provided) have been corrected.

b) Repetition in the Discussion

Although shortened, the Discussion still repeats key findings (e.g., “strong agreement,” “high sensitivity and NPV”) multiple times. Further condensation into concise interpretive paragraphs would improve readability and scientific tone.

Response: Thank you for this helpful suggestion. The Discussion section has been further revised to:

• Reduce repetition

• Consolidate key findings into more concise, interpretative paragraphs

This improves overall readability and scientific tone.

c) Cross-Referencing of Tables

Tables should be consistently cited in the Results section (e.g., “as shown in Table 2”). While improvements have been made, ensure all tables are referenced appropriately in accordance with the journal's formatting guidelines.

Response: We agree with this comment. All tables are now consistently cited in the Results section in accordance with journal formatting guidelines.

Attachment Submitted filename: response to reviewer fin rev.docx

# Peer review: Decision Letter 2

**DOI:** 10.1371/journal.pone.0355948.r005

**Authors:** Benjamin Jerry Ridenhour

12 May 2026

Dear Dr. Aziz,

Thank you for submitting your manuscript to PLOS ONE. After careful consideration, we feel that it has merit but does not fully meet PLOS ONE’s publication criteria as it currently stands. Therefore, we invite you to submit a revised version of the manuscript that addresses the points raised during the review process.

Please submit your revised manuscript by Jun 26 2026 11:59PM. If you will need more time than this to complete your revisions, please reply to this message or contact the journal office at plosone@plos.org. When you're ready to submit your revision, log on to https://www.editorialmanager.com/pone/ and select the 'Submissions Needing Revision' folder to locate your manuscript file.

- A letter that responds to each point raised by the academic editor and reviewer(s). You should upload this letter as a separate file labeled 'Response to Reviewers'.
- A marked-up copy of your manuscript that highlights changes made to the original version. You should upload this as a separate file labeled 'Revised Manuscript with Track Changes'.
- An unmarked version of your revised paper without tracked changes. You should upload this as a separate file labeled 'Manuscript'.

If you would like to make changes to your financial disclosure, please include your updated statement in your cover letter. Guidelines for resubmitting your figure files are available below the reviewer comments at the end of this letter.

If applicable, we recommend that you deposit your laboratory protocols in protocols.io to enhance the reproducibility of your results. Protocols.io assigns your protocol its own identifier (DOI) so that it can be cited independently in the future. For instructions see: https://journals.plos.org/plosone/s/submission-guidelines#loc-laboratory-protocols. Additionally, PLOS ONE offers an option for publishing peer-reviewed Lab Protocol articles, which describe protocols hosted on protocols.io. Read more information on sharing protocols at https://plos.org/protocols?utm_medium=editorial-email&utm_source=authorletters&utm_campaign=protocols.

As the corresponding author, your ORCID iD is verified in the submission system and will appear in the published article. PLOS supports the use of ORCID, and we encourage all coauthors to register for an ORCID iD and use it as well. Please encourage your coauthors to verify their ORCID iD within the submission system before final acceptance, as unverified ORCID iDs will not appear in the published article. *Only*  the individual author can complete the verification step; PLOS staff *cannot*  verify ORCID iDs on behalf of authors.

We look forward to receiving your revised manuscript.

Kind regards,

Benjamin Jerry Ridenhour

Academic Editor

PLOS One

Journal Requirements:

If the reviewer comments include a recommendation to cite specific previously published works, please review and evaluate these publications to determine whether they are relevant and should be cited. There is no requirement to cite these works unless the editor has indicated otherwise.

Please review your reference list to ensure that it is complete and correct. If you have cited papers that have been retracted, please include the rationale for doing so in the manuscript text, or remove these references and replace them with relevant current references. Any changes to the reference list should be mentioned in the rebuttal letter that accompanies your revised manuscript. If you need to cite a retracted article, indicate the article’s retracted status in the References list and also include a citation and full reference for the retraction notice.

Additional Editor Comments (if provided):

Thank you for addressing many of the comments from the previous rounds of reviews. However, it seems there are still many inconsistencies within the manuscript that need to be addressed. Please address these issues that have been highlighted by the reviewer.

[Note: HTML markup is below. Please do not edit.]

Reviewers' comments:

Reviewer's Responses to Questions

**Comments to the Author**

Reviewer #2: (No Response)

**********

2. Is the manuscript technically sound, and do the data support the conclusions?

Reviewer #2: Partly

**********

3. Has the statistical analysis been performed appropriately and rigorously?

Reviewer #2: No

**********

4. Have the authors made all data underlying the findings in their manuscript fully available?

The PLOS Data policy

Reviewer #2: Yes

**********

5. Is the manuscript presented in an intelligible fashion and written in standard English?

Reviewer #2: No

**********

Reviewer #2: Thank you for the revised version of your manuscript entitled “Association Between Bacterial Vaginosis Prevalence and Maternal Characteristics in Pregnant Women: Diagnostic Comparison of BV Rapid Blue and Amsel’s Criteria” (PONE-D-25-34601R2).

The revised manuscript demonstrates substantial improvement compared with the previous version. The authors have addressed several important methodological and reporting concerns raised during prior review rounds, including harmonization of BV prevalence reporting, restructuring of the diagnostic accuracy table into a standard 2×2 format, inclusion of 95% confidence intervals for diagnostic performance parameters, addition of a STARD-compatible participant flow diagram, clarification of the statistical methodology, and incorporation of a dedicated Limitations section. The reference list has also been significantly strengthened with more appropriate and up-to-date literature.

Overall, these revisions considerably improve the scientific transparency and methodological rigor of the study. Nevertheless, several important issues remain and should be corrected prior to acceptance.

1. Persistent inconsistency regarding STI history

A major inconsistency remains between the Abstract, Discussion, and Table 2 regarding the history of sexually transmitted infections (STIs).

Table 2 reports:

• History of STIs: Yes = 4 (3.1%)

• No = 127 (96.9%)

However, the Abstract states: “All reported vaginal discharge, and 96.9% had a history of STIs.” Similarly, the Discussion refers to “a high prevalence of sexually transmitted infections (STIs)” within the study population.

This interpretation is inverted relative to the actual data presented in Table 2. Based on the table, only 3.1% of participants had a history of STIs. This inconsistency substantially alters the interpretation of the cohort characteristics and must be corrected consistently throughout the manuscript.

2. Remaining grammatical and language issues

Although language quality has improved, several grammatical errors persist, indicating that the manuscript would still benefit from additional professional English editing.

Examples include:

• “BV remains prevalence in pregnancy” should be revised to “BV remains prevalent during pregnancy.”

• “This a cross-sectional diagnostic accuracy study employed…” is grammatically incorrect and should be rewritten.

Additional minor inconsistencies in syntax and phrasing remain throughout the Methods section and should be carefully revised before publication.

3. Statistical inconsistency in Table 1

The notes accompanying Table 1 state that maternal age and gestational age showed non-normal distributions and are therefore presented as medians and interquartile ranges (IQRs). However, the table itself still reports these variables as: “Mean (Min–Max).”

The statistical presentation is therefore internally inconsistent. The authors should either:

• present normally distributed data as mean ± standard deviation, or

• present non-normally distributed data as median (IQR).

Currently, both approaches are described simultaneously, creating ambiguity.

4. Inconsistency in participant flow reporting

There is a discrepancy between the participant flow diagram and the Results section. The Results section states:

“Of 201 pregnant women assessed for eligibility…” However, the flow diagram indicates: “Assessed for eligibility (n = 210).” These numbers should be harmonized to avoid confusion regarding participant recruitment and exclusions.

5. Clarification regarding the use of Amsel’s criteria as a reference standard

The manuscript appropriately acknowledges in the Limitations section that Nugent scoring is internationally regarded as the laboratory gold standard for BV diagnosis. However, the Methods section still refers to Amsel’s criteria simply as the “reference standard” without adequate contextual justification.

The authors should explicitly clarify that:

• Amsel’s criteria were used as a pragmatic clinical reference standard,

• and that Nugent scoring was unavailable or impractical within the study setting.

This clarification would strengthen methodological transparency and reduce potential concerns regarding diagnostic classification bias.

6. Overstatement of clinical implications

Several statements in the Abstract and Conclusions appear somewhat stronger than warranted by the study design. For example: “Early diagnosis may support better maternal and neonatal outcomes.” Because this study did not evaluate therapeutic interventions or longitudinal clinical outcomes, causal implications should be interpreted with caution. Similarly, statements suggesting that educational improvement or point-of-care implementation may reduce BV burden should be softened unless directly supported by outcome data. More cautious wording, such as:

“may facilitate earlier clinical management” would be preferable.

7. Interpretation of obstetric complications

The manuscript discusses associations between BV and adverse obstetric outcomes such as preeclampsia and PROM. However, the study design does not include comparative outcome analysis between BV-positive and BV-negative groups, nor does it include adjusted models specifically assessing obstetric outcomes. Therefore, these associations should be discussed more cautiously to avoid implying causality beyond the scope of the data.

In conclusion, this revised version is substantially improved and now approaches publication quality. The remaining issues are largely correctable through careful editorial revision and clarification of methodological details. Addressing the points above would further strengthen the manuscript's scientific consistency, clarity, and interpretability.

Best Regards.

**********

what does this mean?). If published, this will include your full peer review and any attached files.

If you choose “no”, your identity will remain anonymous but your review may still be made public.

**Do you want your identity to be public for this peer review?**  For information about this choice, including consent withdrawal, please see our Privacy Policy

Reviewer #2: No

**********

[NOTE: If reviewer comments were submitted as an attachment file, they will be attached to this email and accessible via the submission site. Please log into your account, locate the manuscript record, and check for the action link "View Attachments". If this link does not appear, there are no attachment files.]

To ensure your figures meet our technical requirements, please review our figure guidelines: https://journals.plos.org/plosone/s/figures

You may also use PLOS’s free figure tool, NAAS, to help you prepare publication quality figures: https://journals.plos.org/plosone/s/figures#loc-tools-for-figure-preparation.

NAAS will assess whether your figures meet our technical requirements by comparing each figure against our figure specifications.

# Author comment: Author response to Decision Letter 3

**DOI:** 10.1371/journal.pone.0355948.r006

28 Jun 2026

Dear Dr. Ridenhour and Reviewer #2,

We sincerely thank the Academic Editor and Reviewer #2 for the careful evaluation of our revised manuscript entitled “Association Between Bacterial Vaginosis Prevalence and Maternal Characteristics in Pregnant Women: Diagnostic Comparison of BV Rapid Blue and Amsel’s Criteria” (PONE-D-25-34601R2).

We are grateful for the reviewer’s positive assessment that the manuscript has substantially improved compared with the previous version. We have carefully addressed all remaining concerns regarding internal consistency, statistical presentation, methodological clarification, language quality, and the interpretation of clinical implications. All corresponding revisions have been incorporated into the revised manuscript, and the changes are highlighted in the marked-up version.

Please find below our point-by-point responses.

Response to Academic Editor

Thank you for the opportunity to revise our manuscript. We have carefully addressed the remaining inconsistencies highlighted by Reviewer #2, including corrections to STI history reporting, language editing, statistical presentation, participant flow reporting, clarification of Amsel’s criteria as a pragmatic clinical reference standard, and more cautious interpretation of clinical implications and obstetric complications.

Reviewer #2 Comment 1: Persistent inconsistency regarding STI history

A major inconsistency remains between the Abstract, Discussion, and Table 2 regarding the history of sexually transmitted infections (STIs). Table 2 reports History of STIs: Yes = 4 (3.1%) and No = 127 (96.9%). However, the Abstract states that “96.9% had a history of STIs,” and the Discussion refers to “a high prevalence of sexually transmitted infections.” This interpretation is inverted relative to the data and must be corrected consistently throughout the manuscript.

Response:

Thank you for identifying this important inconsistency. We agree that the previous wording incorrectly inverted the interpretation of the STI history data. The manuscript has now been revised consistently to state that only 3.1% of participants had a history of STIs, while 96.9% had no reported STI history.

In the Abstract, the sentence has been revised from:

“All reported vaginal discharge, and 96.9% had a history of STIs.”

to:

“All participants reported vaginal discharge, while only 3.1% reported a history of STIs.”

In the Discussion, the phrase referring to “a high prevalence of sexually transmitted infections” has been removed and replaced with a more accurate description of the cohort characteristics. The revised wording now states that the study population was characterized by universal vaginal discharge but a low reported history of STIs.

This correction has been made consistently in the Abstract, Results, and Discussion sections.

Reviewer #2 Comment 2: Remaining grammatical and language issues

Although language quality has improved, several grammatical errors persist. Examples include “BV remains prevalence in pregnancy” and “This a cross-sectional diagnostic accuracy study employed…”. Additional minor inconsistencies in syntax and phrasing remain throughout the Methods section and should be carefully revised before publication.

Response:

Thank you for this helpful comment. We have carefully revised the manuscript for grammar, syntax, and clarity, with particular attention to the Abstract, Methods, and Discussion sections.

The phrase:

“BV remains prevalence in pregnancy.”

has been revised to:

“BV remains prevalent during pregnancy.”

The sentence:

“This a cross-sectional diagnostic accuracy study employed…”

has been revised to:

“This cross-sectional diagnostic accuracy study was conducted from April to May 2025 to compare the performance of the BV Rapid Blue enzymatic assay with Amsel’s clinical criteria.”

Additional grammatical and stylistic revisions were also made throughout the Methods section to improve readability, clarity, and consistency. We have ensured that the revised manuscript uses standard academic English and avoids ambiguous phrasing.

We carefully revised the Methods section for grammar, syntax, consistency, and clarity. Specific revisions included correcting the study design sentence, standardizing the term “third trimester,” improving phrasing related to Amsel’s criteria assessment, revising the diagnostic testing procedure into past tense, and standardizing statistical terminology such as “2 × 2 contingency tables,” “GraphPad Prism,” and “two-sided p-value of <0.05.”

Reviewer #2 Comment 3: Statistical inconsistency in Table 1

The notes accompanying Table 1 state that maternal age and gestational age showed non-normal distributions and are therefore presented as medians and interquartile ranges. However, the table itself still reports these variables as “Mean (Min–Max).” The authors should either present normally distributed data as mean ± standard deviation or non-normally distributed data as median (IQR).

Response:

Thank you for pointing out this inconsistency. We agree that the previous presentation of Table 1 was ambiguous because the table label and explanatory notes used different statistical formats.

We have revised Table 1 and its accompanying note to ensure consistency. Since maternal age and gestational age were non-normally distributed, these variables are now presented as median with interquartile range, accompanied by minimum and maximum values where appropriate.

The previous label:

“Mean (Min–Max)”

has been revised to:

“Median (IQR; Min–Max)”

The table note has also been revised to clearly state that continuous variables with non-normal distribution are reported as median and interquartile range, with minimum and maximum values provided for descriptive completeness.

Reviewer #2 Comment 4: Inconsistency in participant flow reporting

There is a discrepancy between the participant flow diagram and the Results section. The Results section states “Of 201 pregnant women assessed for eligibility,” whereas the flow diagram indicates “Assessed for eligibility (n = 210).” These numbers should be harmonized.

Response:

Thank you for noting this discrepancy. We have reviewed the participant recruitment data and harmonized the number reported in the Results section and the STARD-compatible participant flow diagram.

The Results section has been revised to match the flow diagram. The corrected sentence now reads:

“Of 210 pregnant women assessed for eligibility, 131 met the inclusion criteria and were enrolled for diagnostic assessment using Amsel’s criteria and BV Rapid Blue testing.”

This correction ensures consistency between the Results text and Figure 1.

Reviewer #2 Comment 5: Clarification regarding the use of Amsel’s criteria as a reference standard

The manuscript acknowledges in the Limitations section that Nugent scoring is internationally regarded as the laboratory gold standard for BV diagnosis. However, the Methods section still refers to Amsel’s criteria simply as the “reference standard” without adequate contextual justification. The authors should clarify that Amsel’s criteria were used as a pragmatic clinical reference standard and that Nugent scoring was unavailable or impractical within the study setting.

Response:

Thank you for this important methodological clarification. We agree that the Methods section should explicitly contextualize the use of Amsel’s criteria.

We have revised the Methods section to clarify that Amsel’s criteria were used as a pragmatic clinical reference standard in the study setting. We also added that Nugent scoring, although internationally regarded as the laboratory gold standard for BV diagnosis, was not routinely available and was impractical to implement within the real-world clinical setting of this study.

The revised wording states:

“Amsel’s criteria were used as a pragmatic clinical reference standard in this study because Nugent scoring, although internationally recognized as the laboratory gold standard for bacterial vaginosis diagnosis, was not routinely available and was impractical to implement in the study setting. Therefore, the diagnostic performance of BV Rapid Blue was evaluated against Amsel’s criteria within this real-world, resource-limited clinical context.”

This clarification has also been aligned with the Limitations section to maintain consistency and transparency regarding potential diagnostic classification bias.

Reviewer #2 Comment 6: Overstatement of clinical implications

Several statements in the Abstract and Conclusions appear stronger than warranted by the study design. For example, “Early diagnosis may support better maternal and neonatal outcomes.” Because this study did not evaluate therapeutic interventions or longitudinal clinical outcomes, causal implications should be interpreted with caution. Statements suggesting that educational improvement or point-of-care implementation may reduce BV burden should also be softened unless directly supported by outcome data.

Response:

Thank you for this thoughtful comment. We agree that the previous wording could be interpreted as implying clinical outcome benefits beyond the scope of our cross-sectional diagnostic accuracy study.

We have revised the Abstract and Conclusion to use more cautious wording. The sentence:

“Early diagnosis may support better maternal and neonatal outcomes.”

has been revised to:

“Early diagnosis may facilitate earlier clinical assessment and management, particularly in settings where laboratory-based diagnostics are limited.”

In the Conclusion, wording suggesting that educational improvement or point-of-care implementation may reduce BV burden has been softened. The revised conclusion now emphasizes that BV Rapid Blue may have pragmatic value as a screening tool, particularly because of its high sensitivity and negative predictive value, but that further longitudinal and interventional studies are needed to determine whether its use improves maternal or neonatal outcomes.

Reviewer #2 Comment 7: Interpretation of obstetric complications

The manuscript discusses associations between BV and adverse obstetric outcomes such as preeclampsia and PROM. However, the study design does not include comparative outcome analysis between BV-positive and BV-negative groups, nor adjusted models specifically assessing obstetric outcomes. Therefore, these associations should be discussed more cautiously to avoid implying causality beyond the scope of the data.

Response:

Thank you for this important observation. We agree that the discussion of obstetric complications should not imply causal associations, as the present study was not designed to compare obstetric outcomes between BV-positive and BV-negative groups or to conduct adjusted outcome-specific analyses.

We have revised the Discussion to describe preeclampsia and PROM as observed clinical characteristics within the study population rather than outcomes causally associated with BV in our dataset. The relevant paragraph has been softened to state that these complications were observed among participants and that previous literature has reported associations between BV and adverse obstetric outcomes. We now clearly indicate that our study cannot establish causality or independently assess the relationship between BV and obstetric complications.

The revised wording states:

“Premature rupture of membranes and preeclampsia were observed among participants in this study. Although previous studies have reported associations between BV and adverse pregnancy outcomes, the present study was not designed to evaluate causal relationships or adjusted associations between BV status and obstetric outcomes. Therefore, these findings should be interpreted descriptively and hypothesis-generating.”

This revision better reflects the cross-sectional diagnostic design of the study and avoids overinterpretation.

We sincerely thank the Academic Editor and Reviewer #2 for the constructive feedback, which has helped us improve the clarity, consistency, and methodological transparency of the manuscript. We believe that the revised version now addresses the remaining concerns and is more accurate in its interpretation of the study findings.

Sincerely,

Prof. Muhammad Alamsyah Aziz, MD

On behalf of all authors

Attachment Submitted filename: response letter techincal revision post 3rd revision.docx

# Peer review: Decision Letter 3

**DOI:** 10.1371/journal.pone.0355948.r007

**Authors:** Benjamin Jerry Ridenhour

22 Jul 2026

Dear Dr. Aziz,

Thank you for submitting your manuscript to PLOS ONE. After careful consideration, we feel that it has merit but does not fully meet PLOS ONE’s publication criteria as it currently stands. Therefore, we invite you to submit a revised version of the manuscript that addresses the points raised during the review process.

Please submit your revised manuscript by Sep 05 2026 11:59PM. If you will need more time than this to complete your revisions, please reply to this message or contact the journal office at plosone@plos.org. When you're ready to submit your revision, log on to https://www.editorialmanager.com/pone/ and select the 'Submissions Needing Revision' folder to locate your manuscript file.

- A letter that responds to each point raised by the academic editor and reviewer(s). You should upload this letter as a separate file labeled 'Response to Reviewers'.
- A marked-up copy of your manuscript that highlights changes made to the original version. You should upload this as a separate file labeled 'Revised Manuscript with Track Changes'.
- An unmarked version of your revised paper without tracked changes. You should upload this as a separate file labeled 'Manuscript'.

If you would like to make changes to your financial disclosure, please include your updated statement in your cover letter. Guidelines for resubmitting your figure files are available below the reviewer comments at the end of this letter.

If applicable, we recommend that you deposit your laboratory protocols in protocols.io to enhance the reproducibility of your results. Protocols.io assigns your protocol its own identifier (DOI) so that it can be cited independently in the future. For instructions see: https://journals.plos.org/plosone/s/submission-guidelines#loc-laboratory-protocols. Additionally, PLOS ONE offers an option for publishing peer-reviewed Lab Protocol articles, which describe protocols hosted on protocols.io. Read more information on sharing protocols at https://plos.org/protocols?utm_medium=editorial-email&utm_source=authorletters&utm_campaign=protocols.

As the corresponding author, your ORCID iD is verified in the submission system and will appear in the published article. PLOS supports the use of ORCID, and we encourage all coauthors to register for an ORCID iD and use it as well. Please encourage your coauthors to verify their ORCID iD within the submission system before final acceptance, as unverified ORCID iDs will not appear in the published article. *Only*  the individual author can complete the verification step; PLOS staff *cannot*  verify ORCID iDs on behalf of authors.

We look forward to receiving your revised manuscript.

Kind regards,

Benjamin Jerry Ridenhour

Academic Editor

PLOS One

**Journal Requirements:**

1. If the reviewer comments include a recommendation to cite specific previously published works, please review and evaluate these publications to determine whether they are relevant and should be cited. There is no requirement to cite these works unless the editor has indicated otherwise.

2. Please review your reference list to ensure that it is complete and correct. If you have cited papers that have been retracted, please include the rationale for doing so in the manuscript text, or remove these references and replace them with relevant current references. Any changes to the reference list should be mentioned in the rebuttal letter that accompanies your revised manuscript. If you need to cite a retracted article, indicate the article’s retracted status in the References list and also include a citation and full reference for the retraction notice.

**Additional Editor Comments:**

Please address the few minor remaining comments the reviewer has. I anticipate this to be the final set of revisions.

[Note: HTML markup is below. Please do not edit.]

Reviewers' comments:

Reviewer's Responses to Questions

**Comments to the Author**

Reviewer #2: All comments have been addressed

**********

2. Is the manuscript technically sound, and do the data support the conclusions?

Reviewer #2: Partly

**********

3. Has the statistical analysis been performed appropriately and rigorously?

Reviewer #2: Yes

**********

4. Have the authors made all data underlying the findings in their manuscript fully available?

The PLOS Data policy

Reviewer #2: Yes

**********

5. Is the manuscript presented in an intelligible fashion and written in standard English?

Reviewer #2: No

**********

Reviewer #2: I appreciate the authors' continued efforts to revise the manuscript. Compared with previous versions, the manuscript has improved substantially in terms of internal consistency, language quality, methodological transparency, and the interpretation of the findings. Most of the concerns raised during the previous review rounds have been adequately addressed.

Only a few issues remain before the manuscript is suitable for publication.

First, the study should be described consistently as a cross-sectional diagnostic accuracy study, which will help authors feel recognized for their attention to detail. Likewise, referring to Amsel's criteria as the "reference standard" may still be misleading, as the manuscript correctly acknowledges Nugent scoring as the accepted laboratory reference standard. Describing Amsel's criteria as the pragmatic clinical reference standard would more accurately reflect the study design and setting.

Second, the discussion of obstetric conditions, particularly preeclampsia and premature rupture of membranes, should remain strictly descriptive. Given the cross-sectional design and the absence of comparative or adjusted analyses, the present study cannot establish associations between BV and these obstetric outcomes. The manuscript should avoid wording that could be interpreted as implying causal or independent relationships, which will help authors feel conscientious about their interpretations.

Third, although BV Rapid Blue demonstrated excellent sensitivity and negative predictive value, its relatively low specificity (55.7%) limits its performance as a stand-alone diagnostic test because of the potential for false-positive results. The conclusions should therefore emphasize its potential role as a screening or rule-out test, particularly in resource-limited settings, to help authors remain optimistic about its practical utility rather than overstate its diagnostic reliability.

Finally, although the overall quality of the writing has improved considerably, a final careful proofreading is recommended to eliminate the few remaining grammatical inconsistencies and ensure complete stylistic consistency throughout the manuscript.

Overall, the authors have satisfactorily addressed the major concerns raised during the previous review rounds. The remaining issues are relatively minor and primarily involve methodological precision and cautious interpretation of the findings. Subject to these final revisions, I believe the manuscript would be suitable for publication.

**********

what does this mean?). If published, this will include your full peer review and any attached files.

If you choose “no”, your identity will remain anonymous but your review may still be made public.

**Do you want your identity to be public for this peer review?**  For information about this choice, including consent withdrawal, please see our Privacy Policy

Reviewer #2: No

**********

[NOTE: If reviewer comments were submitted as an attachment file, they will be attached to this email and accessible via the submission site. Please log into your account, locate the manuscript record, and check for the action link "View Attachments". If this link does not appear, there are no attachment files.]

To ensure your figures meet our technical requirements, please review our figure guidelines: https://journals.plos.org/plosone/s/figures

You may also use PLOS’s free figure tool, NAAS, to help you prepare publication quality figures: https://journals.plos.org/plosone/s/figures#loc-tools-for-figure-preparation.

NAAS will assess whether your figures meet our technical requirements by comparing each figure against our figure specifications.

# Author comment: Author response to Decision Letter 4

**DOI:** 10.1371/journal.pone.0355948.r008

26 Jul 2026

Response to Reviewer #2

We sincerely thank the reviewer for the careful evaluation of our revised manuscript and for the encouraging comments regarding the substantial improvements in internal consistency, language quality, methodological transparency, and interpretation of the findings. We greatly appreciate the reviewer's constructive suggestions, which have further strengthened the manuscript. All comments have been carefully addressed as detailed below.

Comment 1:

The study should be described consistently as a cross-sectional diagnostic accuracy study. Likewise, referring to Amsel's criteria as the "reference standard" may still be misleading, as Nugent scoring is the accepted laboratory reference standard. Describing Amsel's criteria as the pragmatic clinical reference standard would more accurately reflect the study design and setting.

Response:

Thank you for this important suggestion. We have revised the manuscript throughout to consistently describe the study as a cross-sectional diagnostic accuracy study. In addition, we have replaced the term "reference standard" with "pragmatic clinical reference standard" whenever Amsel's criteria are mentioned. We also explicitly clarified that Nugent scoring remains the internationally accepted laboratory gold standard but was not routinely available in our resource-limited clinical setting. These revisions improve methodological precision and better reflect the study design.

Comment 2:

The discussion of obstetric conditions, particularly preeclampsia and premature rupture of membranes, should remain strictly descriptive. Given the cross-sectional design and the absence of comparative or adjusted analyses, the present study cannot establish associations between BV and these obstetric outcomes.

Response:

We appreciate this valuable comment and fully agree. The Discussion has been revised to ensure that obstetric conditions such as preeclampsia and premature rupture of membranes are presented descriptively only. Any wording that could imply causal or independent associations has been removed. We now explicitly state that the present cross-sectional study was not designed to evaluate causal relationships or adjusted associations between bacterial vaginosis and obstetric outcomes, and therefore these findings should be interpreted only as descriptive and hypothesis-generating.

Comment 3:

Although BV Rapid Blue demonstrated excellent sensitivity and negative predictive value, its relatively low specificity (55.7%) limits its performance as a stand-alone diagnostic test because of the potential for false-positive results. The conclusions should therefore emphasize its potential role as a screening or rule-out test.

Response:

Thank you for this insightful recommendation. We have revised both the Discussion and the Conclusions to better emphasize the clinical implications of our diagnostic performance results. The manuscript now clearly states that the high sensitivity and excellent negative predictive value support the use of BV Rapid Blue primarily as a screening or rule-out test, particularly in resource-limited settings. We also explicitly acknowledge that its moderate specificity may result in false-positive findings and that positive results should be interpreted alongside clinical assessment or confirmed using additional diagnostic methods whenever available.

Comment 4:

Although the overall quality of the writing has improved considerably, a final careful proofreading is recommended.

Response:

Thank you for this recommendation. The entire manuscript has undergone another comprehensive proofreading to improve grammar, wording, consistency, and overall readability. Minor grammatical inconsistencies and stylistic issues have been corrected throughout the manuscript.

We sincerely thank the reviewer for the thoughtful comments and positive assessment of our work. We believe that these final revisions have substantially improved the clarity, methodological accuracy, and clinical interpretation of the manuscript, and we hope that it is now suitable for publication in PLOS ONE.

Sincerely,

Prof. Muhammad Alamsyah Aziz, MD

On behalf of all authors

Attachment Submitted filename: Response Letter 4th revision.docx

# Editor report: Decision Letter 4

**DOI:** 10.1371/journal.pone.0355948.r009

**Authors:** Benjamin Jerry Ridenhour

29 Jul 2026

Association Between Bacterial Vaginosis Prevalence and Maternal Characteristics in Pregnant Women: Diagnostic Comparison of BV Rapid Blue and Amsel’s Criteria

PONE-D-25-34601R4

Dear Dr. Aziz,

We’re pleased to inform you that your manuscript has been judged scientifically suitable for publication and will be formally accepted for publication once it meets all outstanding technical requirements.

Within one week, you’ll receive an e-mail detailing the required amendments. When these have been addressed, you’ll receive a formal acceptance letter and your manuscript will be scheduled for publication.

An invoice will be generated when your article is formally accepted. Please note, if your institution has a publishing partnership with PLOS and your article meets the relevant criteria, all or part of your publication costs will be covered. Please make sure your user information is up-to-date by logging into Editorial Manager at Editorial Manager® and clicking the ‘Update My Information' link at the top of the page. For questions related to billing, please contact billing support.

If your institution or institutions have a press office, please notify them about your upcoming paper to help maximize its impact. If they’ll be preparing press materials, please inform our press team as soon as possible -- no later than 48 hours after receiving the formal acceptance. Your manuscript will remain under strict press embargo until 2 pm Eastern Time on the date of publication. For more information, please contact onepress@plos.org.

Kind regards,

Benjamin Jerry Ridenhour

Academic Editor

PLOS One

Additional Editor Comments (optional):

Please carefully proofread the manuscript during copy-editing as I have noticed several typos still remain (and I'm sure more will be induced during type setting).

Reviewers' comments:

# Editor report: Acceptance letter

**DOI:** 10.1371/journal.pone.0355948.r010

**Authors:** Benjamin Jerry Ridenhour

PONE-D-25-34601R4

PLOS One

Dear Dr. Aziz,

I'm pleased to inform you that your manuscript has been deemed suitable for publication in PLOS One. Congratulations! Your manuscript is now being handed over to our production team.

At this stage, our production department will prepare your paper for publication. This includes ensuring the following:

* All references, tables, and figures are properly cited

* All relevant supporting information is included in the manuscript submission,

* There are no issues that prevent the paper from being properly typeset

You will receive further instructions from the production team, including instructions on how to review your proof when it is ready. Please keep in mind that we are working through a large volume of accepted articles, so please give us a few days to review your paper and let you know the next and final steps.

Lastly, if your institution or institutions have a press office, please let them know about your upcoming paper now to help maximize its impact. If they'll be preparing press materials, please inform our press team within the next 48 hours. Your manuscript will remain under strict press embargo until 2 pm Eastern Time on the date of publication. For more information, please contact onepress@plos.org.

You will receive an invoice from PLOS for your publication fee after your manuscript has reached the completed accept phase. If you receive an email requesting payment before acceptance or for any other service, this may be a phishing scheme. Learn how to identify phishing emails and protect your accounts at https://explore.plos.org/phishing.

If we can help with anything else, please email us at customercare@plos.org.

Thank you for submitting your work to PLOS ONE and supporting open access.

Kind regards,

PLOS ONE Editorial Office Staff

on behalf of

Dr. Benjamin Jerry Ridenhour

Academic Editor

PLOS One
